# Tritium separation from gaseous ^1,2,3^H isotopologue mixtures by selective adsorption on Ag-exchanged zeolite type Y

**DOI:** 10.1038/s41467-026-75930-9

**Published:** 2026-07-27

**Authors:** Alexandra Becker, Holger Lippold, Jing Liu, Michael Hirscher, Cornelius Fischer

**Affiliations:** 1 Helmholtz-Zentrum Dresden-Rossendorf, Institute of Resource Ecology, Department of Reactive Transport, Leipzig, Germany; 2 Leipzig University, Faculty of Chemistry, Leipzig, Germany; 3 Max Planck Institute for Solid State Research, Stuttgart, Germany; 4 Advanced Institute for Materials Research (WPI-AIMR), Tohoku University, Sendai, Japan

**Keywords:** Porous materials, Nuclear fusion and fission

## Abstract

Efficient separation of hydrogen isotopologues is crucial for applications such as the recycling of exhaust streams in nuclear fusion reactors. We report on separation of a ternary ^1,2,3^H isotope mixture using thermal desorption spectroscopy (TDS), achieving an enrichment of 1:41:175 (H_2_:D_2_:T_2_) from an initially equimolar (1:1:1) gas mixture, based on selective adsorption using an Ag(I)-exchanged zeolite type Y. Further experiments on binary hydrogen isotope mixtures validated numerical predictions of the separation efficiency for T_2_. Specifically, the high selectivity for tritium over protium of 244 makes the Ag(I)-exchanged zeolite an excellent candidate for energy-efficient isotope separation at liquid-nitrogen temperature.

## Abstract (web summary)

Tritium can be separated from binary and ternary isotope gas mixtures via selective adsorption on porous media. The authors report high tritium separation efficiency using Ag(1)-exchanged zeolite Y at temperatures above that of liquid nitrogen.

## Introduction

Among the CO_2_-free energy options, nuclear fusion is regarded as particularly promising and is being pursued in large-scale projects such as the International Thermonuclear Experimental Reactor (ITER)^1^. The reaction fuel consists of the hydrogen isotopes deuterium and tritium. However, only about 2% of the fuel is consumed and therefore, a continuous recycling by isotope separation is required. The exhaust stream additionally contains protium due to secondary neutron-induced reactions and outgassing from structural materials^2,3^. Consequently, the materials and technologies used for isotope separation in fusion power plants must exhibit high selectivity for ternary hydrogen isotope mixtures and, in addition, radiation stability.

Currently, tritium is used in self-powered illumination technologies, for instance in watches and aviation instruments. In these applications, ultra-thin glass capillaries are filled with tritium gas, where β-particles from nuclear decay excite a luminescent coating to produce light^4^. Recycling and reuse of such devices necessitate efficient purification and separation processes, particularly to recover tritium from H_2_.

Cryogenic distillation is an established method for separating the stable isotopes hydrogen and deuterium^5^. However, analyses indicate that this technique is not well suited for the broad range of compositions expected in fusion reactor processes^6^. In recent years, alternative ways have been developed, particularly adsorption-based separation using microporous materials such as metal-organic frameworks (MOFs) or zeolites.

In microporous materials, two quantum effects can be exploited for the separation of hydrogen isotopologues: kinetic quantum sieving (KQS) and chemical affinity quantum sieving (CAQS), both governed by the pore structure and chemical composition of the adsorbent material^7^. KQS, first described by Beenakker et al.^8^ in 1995, arises from differences in the effective particle size (de Broglie wavelength) of the isotopologues, resulting in preferential adsorption of the heavier isotope^9,10^. A variety of crystalline materials with chemically tunable cavities are currently being investigated to elucidate the KQS mechanisms and to optimize separation efficiency^11^. CAQS, in contrast, relies on strong adsorption sites^12,13^. The isotopologues exhibit distinct adsorption enthalpies, determined by the mass-dependent zero-point energy (ZPE) in the van der Waals potential, which allows for quantum sieving on strong binding sites even at temperatures above 80 K^14–16^.

Various classes of porous materials have been experimentally investigated for the separation of the stable hydrogen isotopes H_2_ and D_2_^14,17–19^. However, temperature-resolved studies involving T_2_ are missing. Separation factors have so far mostly been predicted theoretically, based on calculations consistent with experimental H_2_/D_2_ separation data. The calculations predict selectivities of *S*_T/H_ = 40.6 and *S*_T/D_ = 3.5 for 1:1 binary gas mixtures at 80 K on Ag-exchanged ZSM-5 zeolite^20^, with selectivities based on the molar ratios of the adsorbed amounts.

For radioactive tritium, the structural stability of microporous materials needs critical consideration. Zeolites are particularly promising for tritium separation, having demonstrated structural resilience under varying radiation doses and tolerance to tritium decay^21,22^. Open questions remain regarding the radiolytic resistance of other microporous materials considered for separation applications, such as MOFs^23^, as well as of two-dimensional layered materials like graphene, particularly with respect to structural modifications^24^.

Thermal desorption spectroscopy (TDS)^7^ is the method of choice for investigating the adsorption and desorption of hydrogen isotopologues under temperature-controlled conditions. It enables direct sample activation within the setup and operates over a wide temperature range, starting at cryogenic conditions. To date, TDS studies on tritium have not been conducted, as handling highly radioactive gases requires specialized analytical solutions that comply with safety regulations.

In this study, we validate the predicted separation factors for hydrogen isotope mixtures that include tritium by employing the TDS technique with a setup similar to Zhang et al.^16^, additionally equipped with a uranium tritide source in a vacuum compartment system designed for the preparation of gas mixtures including all hydrogen isotopes, and with a flow reactor converting desorbed tritium into HTO for analysis and safe disposal (Supplementary Fig. 1). As a microporous adsorbent substrate, an Ag(I)-exchanged zeolite type Y (AgY) was used. This allows for a direct comparison with a previous study on protium-deuterium mixtures, where the validity of the CAQS mechanism at strong Ag sites has been demonstrated by DFT calculations. Physisorption on sites other than Ag does not occur above liquid-nitrogen temperature^16^.

## Results

### Separation of a ternary hydrogen isotopologue mixture

**Fig. 1:**
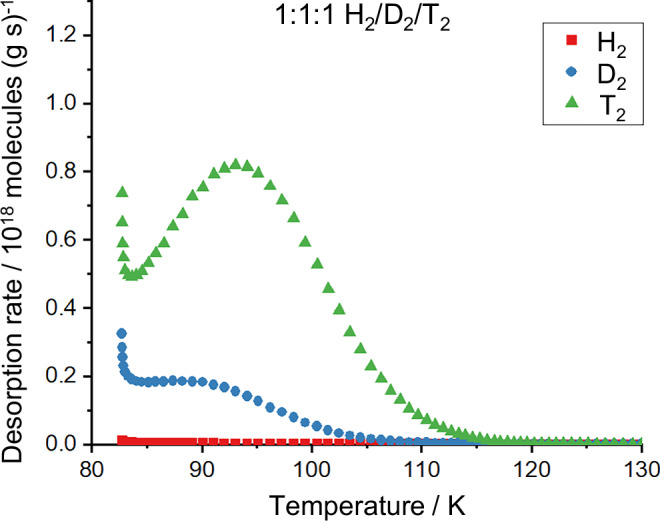
Desorption spectra of a ternary 1:1:1 hydrogen isotopologue mixture. Desorption rates for H_2_ (red), D_2_ (blue) and T_2_ (green) during heating at 0.1 K s^−1^ after adsorption on AgY zeolite for 10 min at ~83 K. The equimolar mixture was introduced at a total pressure of 1 kPa.

Of particular interest is the separation of isotopologues in a ternary mixture relevant for fusion power plants^25^, shown in Fig. 1. The TDS spectra reveal pronounced selectivities, evident from the total amounts desorbed after exposure to an equimolar H_2_/D_2_/T_2_ mixture at 83 K. Tritium adsorption is notably favored, whereas deuterium adsorption is moderate and protium adsorption is almost completely suppressed. The resulting enrichment ratios are 1:41:175 (H_2_:D_2_:T_2_) on a molar basis when solely taking the homonuclear isotopologues into account. All gas compositions, including estimated values for the heteronuclear isotopologues, are given in Supplementary Table 2. The D_2_ measurements by mass spectrometry were minimally affected by HT formation. The total gas uptake was 0.30 mmol g^−1^, which is well below surface saturation^26^.

### Separation of binary hydrogen isotopologue mixtures

**Fig. 2:**
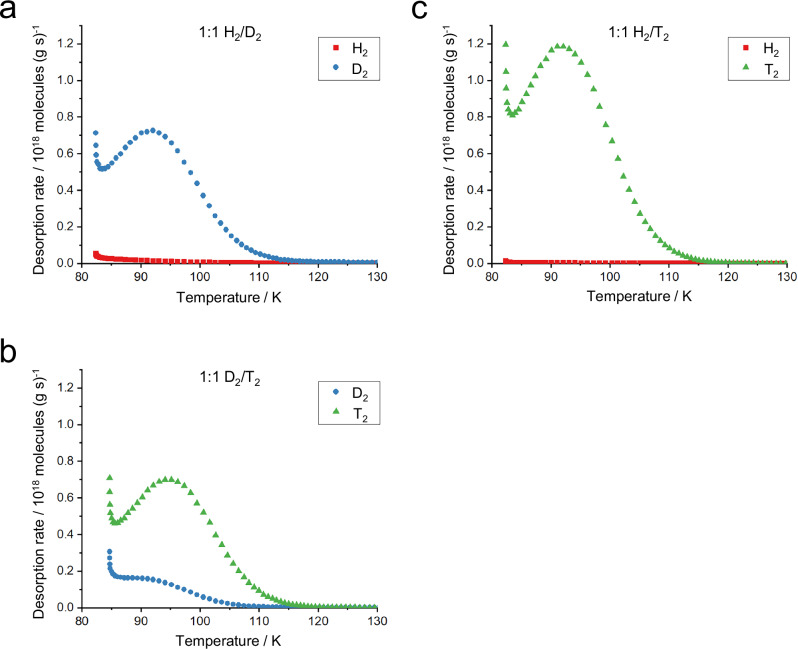
Desorption spectra of binary 1:1 hydrogen isotopologue mixtures. **a** H_2_/D_2_ (adsorption at ~82 K). **b** D_2_/T_2_ (adsorption at ~85 K). **c** H_2_/T_2_ (adsorption at ~82 K). All other conditions correspond to the separation of the ternary system. The equimolar mixtures were introduced at a total pressure of 1 kPa.

TDS analysis of the constituent binary isotopologue mixtures provides insight into the thermodynamic basis of the superimposed selectivities observed in the ternary system. Both the magnitude of gas uptake and the temperature at which maximum desorption rates occur should be considered when characterizing the system’s selective behavior. For an equimolar H_2_/D_2_ mixture (Fig. 2a), a selectivity of *S*_D/H_ = 29 was obtained, exceeding all reported values on protium-deuterium separation achieved by conventional techniques^27^. The total uptake of 0.22 mmol g^−1^ is lower than for the ternary system as the most affinive component is not contained. The D_2_ signal is at maximum at ~92 K, while the H_2_ curve shows no maximum within the measured temperature range. This enables direct quantitative comparison with tritium-containing binary mixtures:

For the D_2_/T_2_ mixture (Fig. 2b), a plateau is observed at ~89 K for D_2_ and a maximum at ~95 K for T_2_. The higher desorption temperatures of the heavier isotopologues confirm their higher adsorption enthalpies, which correlate with their preferential adsorption by CAQS. Moreover, this difference in adsorption energies explains why the uptake ratios in isotopologue mixtures are more pronounced than in the corresponding single-gas systems (Supplementary Fig. 2). During gas exposure, the exchange of isotopologues at adsorption sites initially occupied by lighter isotopes results in uptake ratios that differ from those inferred from single-gas experiments. Competitive adsorption effects are also evident in shifts in the maximum desorption temperatures, as observed for D_2_ in the presence of H_2_ and T_2_. The obtained selectivity of *S*_T/D_ = 4.2, with a total gas uptake of 0.26 mmol g^−1^, is close to the predicted value (*S*_T/D_ = 3.5) for the separation on Ag-exchanged ZSM-5 at 80 K^20^. These values are higher than the T_2_/D_2_ uptake ratio of 2.11 (at 87 K) reported in a previous study using Ag(I)-exchanged zeolite type Y (ref. ^26^), where a cumulative approach was employed for analyzing the gas mixture composition.

For the binary H_2_/T_2_ mixture (Fig. 2c), the total gas uptake was 0.35 mmol g^−1^, with H_2_ contributing minimally. The selectivity has a very high value of *S*_T/H_ = 244. As in the H_2_/D_2_ system, no desorption maximum for H_2_ was detected within the measured temperature range, whereas T_2_ exhibits a maximum at ~91 K.

For all gas mixtures, formation of heteronuclear isotopologues (HD, HT, DT) by isotope exchange was found to be very minor compared to the total adsorbed amounts (Supplementary Table 2). Owing to the strong enrichment of the heaviest isotope in the adsorbed phase, the small fraction of adsorbed H_2_ undergoes an enhanced isotope scrambling. Still, the resulting heteronuclear isotopologues are negligible in relation to the total amount of H_2_ involved in the separation process. The adsorbate compositions suggest that isotope exchange is largely confined to the adsorbed state.

In view of the well-known sensitivity of Ag(I) ions to reduction in consequence of ionizing radiation (used in photo emulsions for dosimetry and autoradiography), the question needs to be addressed as to whether the Ag(I) adsorption sites are affected by the beta radiation. After 4 hours of exposure to T_2_ under the conditions applied in the separation experiments (1 kPa T_2_, 82 K), no significant change in the desorption spectra was detected based on triplicate TDS records for D_2_ before and after T_2_ contact (Supplementary Fig. 5 and Supplementary Table 3). Obviously, the extent of ionization within the zeolite lattice is too low for an electron transfer to the Ag(I) ions. Multiple exposures to T_2_-containing gas mixtures will not affect the separation performance.

We present an efficient method for separating tritium from binary and ternary isotope mixtures by selective adsorption on an Ag(I)-exchanged zeolite Y. This straightforward process exploits the stronger interaction of hydrogen isotopologues with Ag^+^ cations in the micropores. Owing to the high affinity, the required cryogenic temperatures can be achieved using liquid nitrogen, which offers a significant energy advantage over conventional methods such as cryogenic distillation. For binary, equimolar isotope mixtures, high selectivities of 29, 244, and 4.2 have been determined for D_2_/H_2_, T_2_/H_2_, and T_2_/D_2_, respectively, achieved by chemical affinity quantum sieving on Ag^+^ sites. Furthermore, a negligible isotope exchange is observed relative to the total quantity of exposed gas, and the AgY zeolite proves to be radiation-resistant, making it a potential material for efficient tritium enrichment in large-scale technological facilities.

## Methods

### Sample preparation

For the separation experiments, Ag(I)-exchanged zeolite type Y (AgY) was used, which was prepared at Max-Planck-Institut für Kohlenforschung (Mülheim an der Ruhr, Germany) by ion exchange on NaY zeolite (Clariant, Germany) with AgNO_3_. Elemental analysis by ICP-OES indicated complete replacement of Na by Ag (ref. ^16^). A 1.7 mg sample was activated under vacuum in the TDS at 500 K for 2 h to desorb H_2_O and other components.

### Gases

H_2_ with a purity of 99.9992% was supplied by Air Products (Germany), D_2_ with a purity of 99.8% was purchased from Air Liquide (Germany). T_2_ supply was realized using the uranium getter technology^28^, which is based on reversible thermal desorption of a UT_3_ source (3.7 TBq), conducted in a stainless-steel vacuum compartment system (RC TRITEC AG, Switzerland), also allowing for the preparation of gas mixtures. T_2_ gas is set free at a temperature of ~400 °C and is completely rebound by chemisorption at room temperature. Before T_2_ dosing, the decay product ^3^He was baked out of the uranium bed in a heating-cooling sequence.

### Thermal desorption spectroscopy and tritium capture

A quadrupole mass spectrometer (PrismaPro QMG 250, Pfeiffer Vacuum, Germany) was used for monitoring gas desorption rates. Calibration for H_2_ and D_2_ was performed via weight determination using a Pd_98_Ce_2_ alloy^16^. For T_2_, a different approach was needed for safe handling. T_2_ desorbed in a TDS run was purged with air flow from the outlet of the mass spectrometer pump to a reactor tube where it was converted to HTO over CuO catalyst (Merck, Germany) at 910 °C. The reaction product was collected in a water trap, which was sampled for analysis by liquid scintillation counting (LSC) using a Tri-Carb 3110 TR instrument (Perkin Elmer, US) and Ultima Gold™ scintillation cocktail (Perkin Elmer, US), providing a basis for a radiometric calibration. The completeness of conversion and trapping was recently demonstrated^29^. The entire setup is shown in Supplementary Fig. 1.

In preparation of the separation experiments, 1.7 mg zeolite was exposed to the pure isotopologue gases to characterize their adsorption and desorption behavior. For this, the sample chamber was flooded with 1 kPa of the gases at room temperature. The sample was cooled down using liquid N_2_ to temperatures as low as approx. 82 K. After equilibration for 10 min, the gas phase was removed by evacuation (in the case of H_2_ and D_2_) or by sorption onto uranium (in the case of T_2_). Gas mixtures, prepared immediately before the separation experiments, were introduced after cooling, using the same equilibration time and a total pressure of 1 kPa (at room temperature). For mixtures containing T_2_, a separate uranium bed was used for gas removal. A linear temperature program was run up to 300 K at a heating rate of 0.1 K s^−1^ while measuring the desorption rates by mass spectrometry. The integrals of the desorption spectra, corresponding to the adsorbed amounts, were calculated based on the entire temperature range.

### Reporting summary

Further information on research design is available in the Nature Portfolio Reporting Summary linked to this article.

## Supplementary information

Supplementary Information

Reporting Summary

Transparent Peer Review file

## Data Availability

Source data are provided with this paper, available on the data repository RODARE under the 10.14278/rodare.4644.
