# Supplementary material for: Tritium separation from gaseous 1,2,3H isotopologue mixtures by selective adsorption on Ag-exchanged zeolite type Y
Source: Nat Commun. 2026 Jul 27;17:7405. doi: 10.1038/s41467-026-75930-9 (PMC13408095; doi:10.1038/s41467-026-75930-9)
Supplement: Supplementary file 1 — Supplementary Information [file 41467_2026_75930_MOESM1_ESM.pdf]

Supplementary Information

Tritium separation from $^{1,2,3}H$ isotopologue mixtures by selective adsorption on Ag-exchanged zeolite type Y

Alexandra Becker[1,2]*, Holger Lippold[1], Jing Liu[1,2], Michael Hirscher[3,4]*, Cornelius Fischer[1,2]

[1]Helmholtz-Zentrum Dresden-Rossendorf, Institute of Resource Ecology, Department of Reactive Transport, Leipzig, Germany.

[2]Leipzig University, Faculty of Chemistry, Leipzig, Germany.

[3]Max Planck Institute for Solid State Research, Stuttgart, Germany.

[4]Advanced Institute for Materials Research (WPI-AIMR), Tohoku University, Sendai, Japan.

Table of contents

1. Preparation of Ag(I)-exchanged zeolite 3

2. Experimental setup 4

Supplementary Fig. 1. Schematic of the experimental setup. 4

3. TDS data for single component systems 5

Supplementary Fig. 2. Desorption spectra of the individual hydrogen isotopologues. 5

Supplementary Fig. 3. Desorption spectra of the individual hydrogen isotopologues (repetition experiments). 6

Supplementary Table 1. Gas uptakes and rate maximum positions taken from the desorption spectra of the individual hydrogen isotopologues. 6

4. Additional TDS data for isotopologue mixtures 7

Supplementary Fig. 4. Desorption spectra of a second 1:1 D_2/T_2 mixture. 7

Supplementary Table 2. Desorbed amounts of all isotopologues in binary and ternary mixtures. 8

5. Effect of T_2 exposure on desorption profiles and uptakes 9

Supplementary Fig. 5. Desorption spectra of D_2 before and after prolonged exposure of Ag(I)-exchanged zeolite to T_2. 9

Supplementary Table 3. Total uptakes of D_2 on Ag(I)-exchanged zeolite before and after prolonged exposure to T_2. 9

6. References 10

1. Preparation of Ag(I)-exchanged zeolite

For ion exchange, 1 g of zeolite NaY (Clariant, Germany) was treated in 125 mL of a 0.1 M $AgNO_3$ solution (2.12 g of $AgNO_3$ dissolved in 125 mL of deionized argon-saturated water) for 2 h under an Ar atmosphere in a vessel protected from light. The material was then separated by vacuum filtration and was washed with deionized argon-saturated water. The procedure was repeated another two times. The final product was dried at 80°C for 12 h and was stored in the absence of light.

The elemental composition was determined by ICP-OES after digestion. The Ag/Al ratio was found to be 1.04, indicating that Na was completely exchanged for Ag, occupying all Al sites. The residual Na content was below the detection limit of 2.6 ng g^{-1}. Previous studies by XRD have shown that the zeolite Y structure is well preserved after ion exchange. The water content was 15.1 wt.% as determined by TG/DSC.[1]

2. Experimental setup

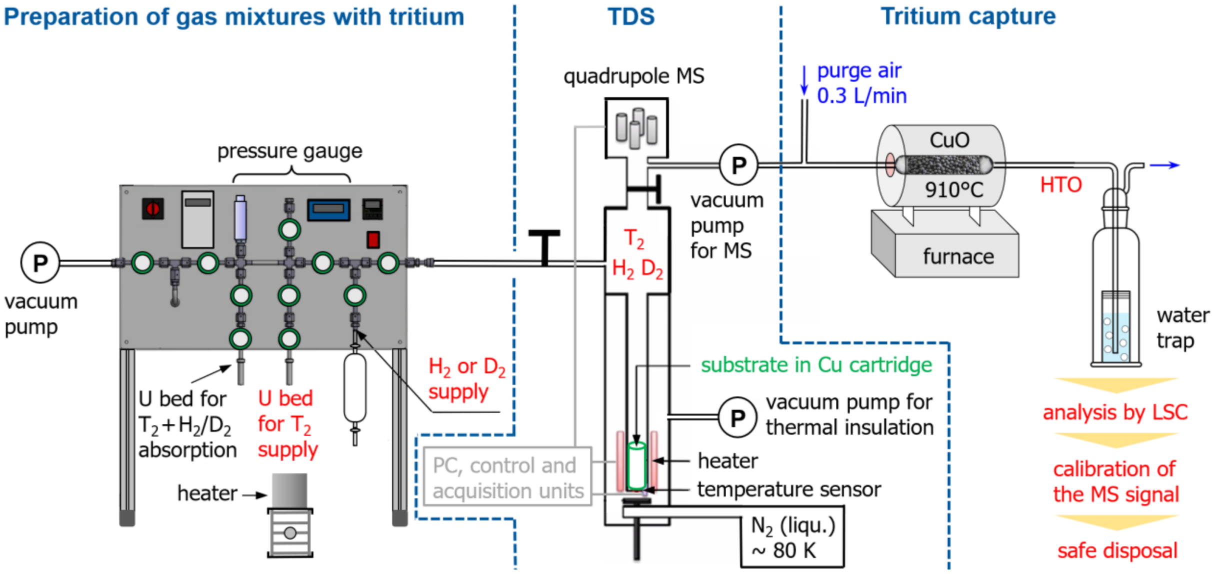

Supplementary Figure 1. **Schematic of the experimental setup.** Left: Vacuum compartment system with UT_3 source. Middle: Setup for thermal desorption spectroscopy (TDS). Right: Flow reactor for tritium capture.

3. TDS data for single component systems

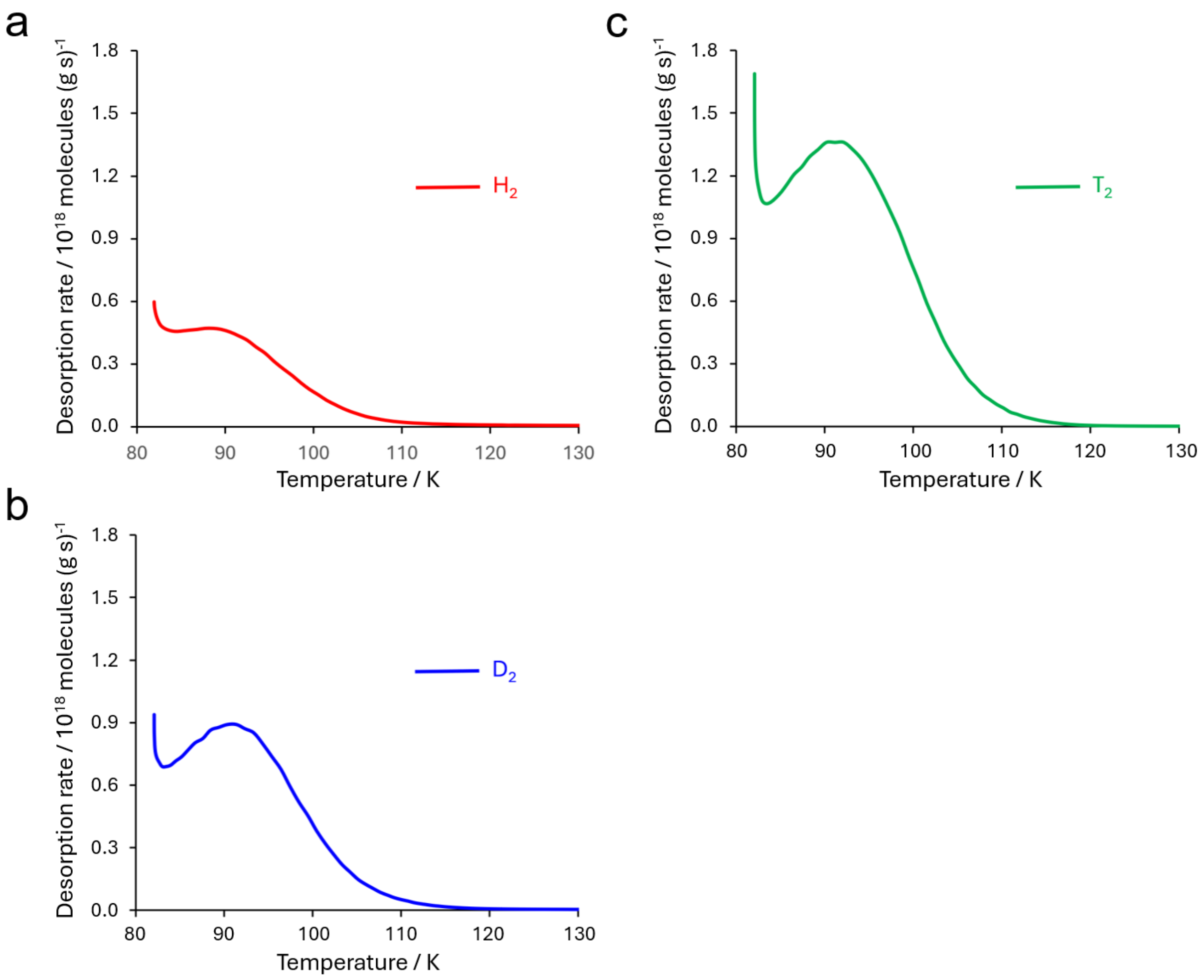

Supplementary Figure 2. **Desorption spectra of the individual hydrogen isotopologues.** Desorption rates during heating at 0.1 K s^{-1} after adsorption on AgY zeolite at ~ 82 K for 10 min. **a** Desorption spectrum of H_2. **b** Desorption spectrum of D_2. **c** Desorption spectrum of T_2. The gases were introduced at a pressure of 1 kPa.

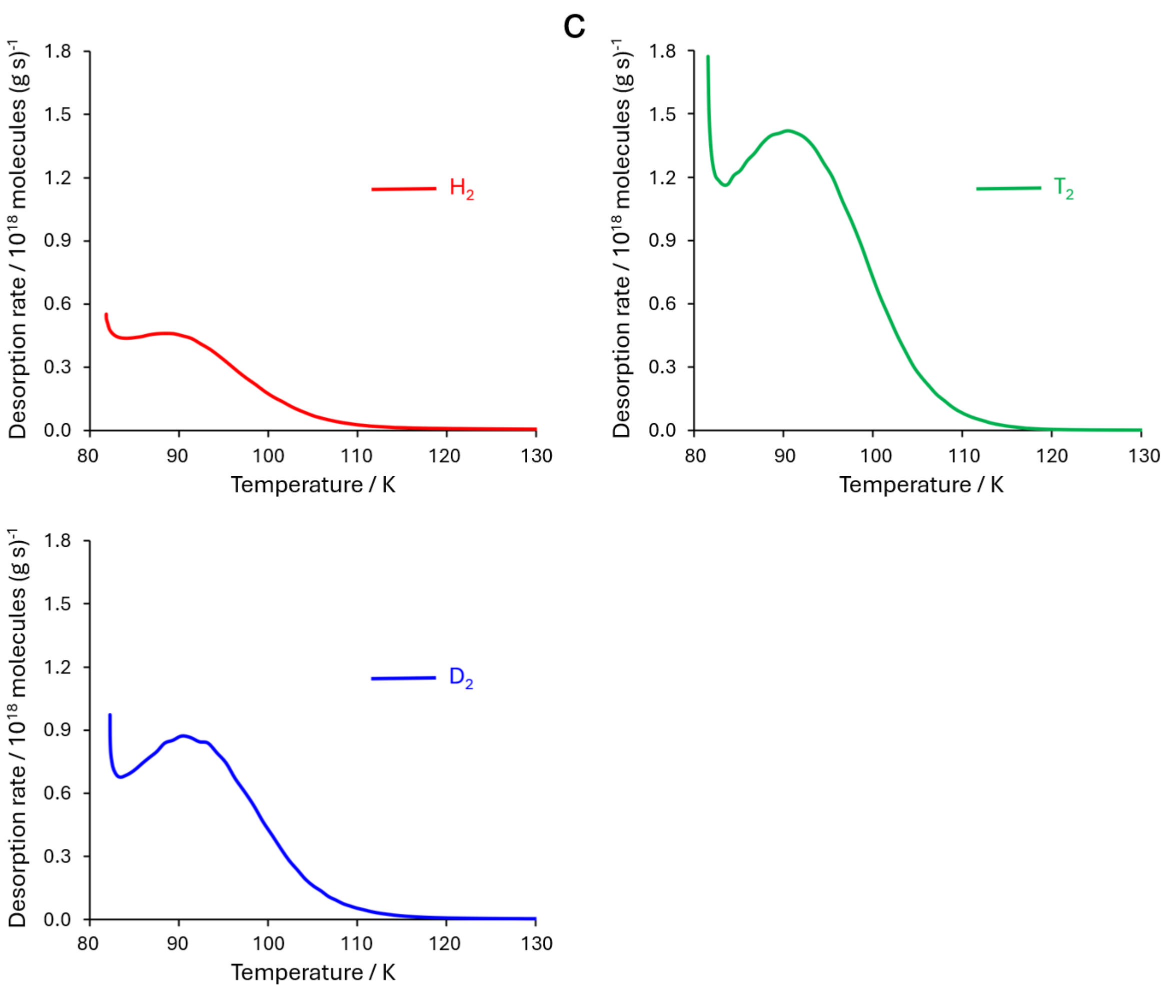

Supplementary Figure 3. **Desorption spectra of the individual hydrogen isotopologues (repetition experiments).** Experimental conditions as indicated in Supplementary Fig. 2. **a** Desorption spectrum of H_2. **b** Desorption spectrum of D_2. **c** Desorption spectrum of T_2.

Supplementary Table 1. **Gas uptakes and rate maximum positions taken from the desorption spectra of the individual hydrogen isotopologues.**

	**1st measurement**		**2nd measurement**	
**Gas**	**Total uptake** (mmol g^{-1})	**Maximum** (K)	**Total uptake** (mmol g^{-1})	**Maximum** (K)
H_2	0.147	88.2	0.145	88.5
D_2	0.260	90.8	0.259	90.3
T_2	0.410	91.1	0.424	90.5

The maximum positions are slightly shifted to higher temperatures for the heavier isotopologues, in accordance with their stronger adsorption visible from the total uptakes. The uptakes were determined by integrating the desorption spectra starting from the exposure temperature.

4. Additional TDS data for isotopologue mixtures

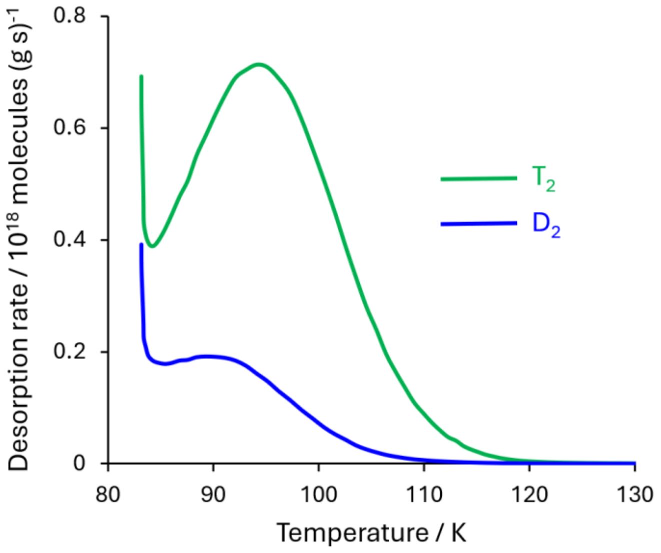

Supplementary Figure 4. **Desorption spectra of a second 1:1 D_2/T_2 mixture.** Desorption rates for D_2 (blue) and T_2 (green) during heating at 0.1 K s^{-1} after adsorption on AgY zeolite for 10 min at ~ 83 K. The mixture was introduced at a total pressure of 1 kPa. The calculated selectivity $S_{T/D}$ is 3.3.

Supplementary Table 2. **Desorbed amounts of all isotopologues in binary and ternary mixtures.**

**System**	**Exposure temperature** (K)	**Amount** (mmol g^{-1})						
		**H_2**	**HD**	**HT**	**D_2**	**DT**	**T_2**	**Total**
H_2/D_2	82.4	0.007	< 0.001	–	0.211	–	–	0.219
H_2/T_2	82.3	0.001	–	0.003	–	–	0.344	0.348
D_2/T_2 (1)	84.7	–	–	–	0.048	< 0.007	0.201	0.256
D_2/T_2 (2)	83.2	–	–	–	0.066	< 0.006	0.219	0.291
$H_2/D_2/T_2$	82.8	0.001	< 0.002	unknown	0.055	< 0.004	0.236	0.298

Desorbed amounts of HD, HT and DT were calculated from the MS data using calibration factors derived by averaging the calibration factors of the neighboring homonuclear isotopologues, based on a linear correlation. For HD and DT, an overlap by asymmetric peak tailing extending from $m/z = 4$ and $m/z = 6$, respectively, had to be taken into account. For a conservative estimate on the maximum contributions of isotope exchange, a lower bound of the tailing contributions was derived from data analyses for single gas systems at varying D_2 and T_2 peak intensities.

The D_2 amount measured for the ternary mixture includes a small unknown amount of HT.

5. Effect of T_2 exposure on desorption profiles and uptakes

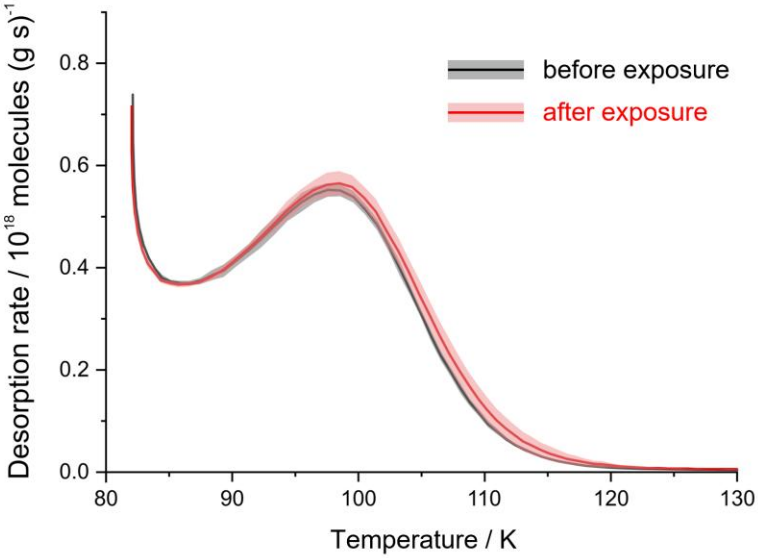

Supplementary Figure 5. **Desorption spectra of D_2 before and after prolonged exposure of Ag(I)-exchanged zeolite to T_2.** Desorption rates during heating at 0.1 K s^{-1} after adsorption at ~ 82 K for 10 min. D_2 was introduced at a pressure of 1 kPa. The lines represent the mean values, the shaded areas show the standard deviations for triplicate TDS records before and after T_2 exposure of the zeolite. For exposure, T_2 was introduced at a pressure of 1 kPa and was left in the adsorption equilibrium at ~ 82 K for 4 h. T_2 was then desorbed in a TDS run. The total number of decays of adsorbed T_2 during exposure was 1.22×10^{16}.

(A pristine sample was used for these investigations, not affected by T_2 exposure in previous TDS experiments.)

Supplementary Table 3. **Total uptakes of D_2 on Ag(I)-exchanged zeolite before and after prolonged exposure to T_2.**

**No. of TDS run**	**Gas uptake** (mmol g^{-1})	
	**before exposure**	**after exposure**
1	0.191	0.212
2	0.195	0.194
3	0.199	0.201
**Mean value:**	0.195	0.202
**Standard deviation:**	0.004	0.009

Significant effects on desorption profiles and uptakes are not observed, demonstrating the material's resilience to the beta radiation.

6. References

1. Zhang, L., Wulf, T., Baum, F., Schmidt, W., Heine, T. & Hirscher, M. Chemical affinity of Ag-exchanged zeolites for efficient hydrogen isotope separation. *Inorg. Chem.* **61**, 9413-9420 (2022).
