# Supplementary material for: Tritium separation from gaseous 1,2,3H isotopologue mixtures by selective adsorption on Ag-exchanged zeolite type Y
Source: Nat Commun. 2026 Jul 27;17:7405. doi: 10.1038/s41467-026-75930-9 (PMC13408095; doi:10.1038/s41467-026-75930-9)
Supplement: Supplementary file 3 — Transparent Peer Review file [file 41467_2026_75930_MOESM3_ESM.pdf]

nature portfolio

Peer Review File

Tritium separation from gaseous $^{1,2,3}H$ isotopologue mixtures by selective adsorption on Ag-exchanged zeolite type Y

Corresponding Author: Dr Michael Hirscher

This file contains all reviewer reports in order by version, followed by all author rebuttals in order by version.

Version 0:

Reviewer comments:

Reviewer #1

(Remarks to the Author)
This (communication type) manuscript reports a timely and consequential experimental study of hydrogen isotope separation on Ag(I)-exchanged zeolite Y that, in my view, merits rapid publication after minor revision. The authors present, to my knowledge, the first 'ternary mixture TDS profiles' that directly resolve tritium within both binary and ternary mixtures and extract quantitative selectivities S_D/H=7.7, S_T/H=38.2, S_T/D=2.5 from integrated TDS signals, while documenting minimal isotope exchange products (HD/HT/DT) and stable total uptake upon repeated T_2 exposure, key indicators of data reliability and material robustness under radiolytic conditions. These findings create a needed experimental benchmark for the fusion fuel-cycle community by demonstrating effective chemical-affinity quantum sieving (CAQS) at liquid-nitrogen-accessible temperatures and by reporting ternary $H_2/D_2/T_2$ outcomes that are directly relevant to process design, including a ternary desorption composition with T_2 at ~66–70% depending on whether mixed isotopologues are counted.

There is, however, one non-trivial inconsistency that the authors should resolve before acceptance. In single-gas TDS, the reported absolute uptake for H_2 (~0.72 mmol g^{-1}) is roughly twice that of D_2 (~0.35 mmol g^{-1}) and T_2 (~0.33 mmol g^{-1}) under nominally similar conditions. In most physisorption systems, the number of molecules occupying a given site is comparable across isotopes (or, if anything, slightly higher for the heavier isotope because of lower zero-point energy) so the present inverse trend needs to be explained. A plausible hypothesis is that a calibration constant slips during the initial hydrogen standardization, specifically treating the signal as "H" (atomic) rather than "H_2" (diatomic), which would inflate the apparent H_2 uptake by about a factor of 2. (Indeed, although it is shown to have double the H2 uptake, the performance of this system will be further enhanced when the calibration constant is corrected.)

Reviewer #2

(Remarks to the Author)
Becker and coworkers have reported in this manuscript the separation of T2 from hydrogen isotopes utilizing Ag-exchanged zeolite Y at temperatures exceeding 77 K. This achievement represents a significant and exciting advancement in the field of hydrogen isotope separation. The manuscript will be deemed appropriate for publication in Nature Communications provided that the following concerns are adequately addressed:

1) The AgY zeolite was synthesized through ion exchange from NaY using AgNO3, as detailed in the manuscript. What is the Ag content in the target AgY? Additionally, will the Ag content influence the T2 separation performance?

2) T2 is a radioactive substance that may potentially damage the structure of the sorbent as mentioned in the manuscript. A comparative analysis of the structural integrity of AgY zeolite before and after exposure to T2 should be conducted, focusing on PXRD, physisorption, and Ag content.

3) Measurements of single-component physisorption for H2, D2, and T2 at temperatures ranging from 80 to 85 K are

required.

4) The complete name of 'ITER' should be presented upon its initial mention in the main text.

5) The ref 4 is incorrectly formatted, and the page number for ref 9 is also incorrect.

Reviewer #3

(Remarks to the Author)
This manuscript describes the separation of $H_2/D_2/T_2$ mixtures using Ag(I)-exchanged zeolite Y, as evaluated by TDS. While the handling of tritium in the experiments is technically challenging and commendable, the study offers limited novelty and fails to provide significant beyond the existing literature. This is particularly evident in light of the comprehensive study by Xiong et al, which has already elucidated the mechanism and selectivity trends for T_2/D_2 separation in Ag^+-exchanged zeolites. Consequently, the work in its current form is not suitable for publication in Nature Communications. The specific concerns are outlined below:
1. The authors must clarify the new fundamental insights or performance advantages offered by this work relative to prior studies. The feasibility, mechanism, and selectivity of Ag^+-exchanged zeolites for separating D_2/H_2 and T_2/D_2 have already been established in the literature (Chemical Engineering Journal, 2024, 493, 152673). Simply extending the application from binary to ternary gas mixtures is considered insufficient to constitute a significant advance.
2. The discussion of the CAQS mechanism remains purely qualitative. To substantiate these claims, it is essential to provide quantitative support, such as through relevant theoretical calculations (e.g., adsorption energies, binding strengths).
3. The manuscript omits key material properties, including Ag^+ loading, cation exchange degree, and site distribution. Given the direct influence of Ag^+ concentration on adsorption enthalpy and isotopic selectivity, this gap critically undermines both the reproducibility of the work and the interpretation of the underlying mechanism.
4. The assertion that the material exhibits radiation stability is not backed by structural characterization data (e.g., XRD, BET, EXAFS). Direct evidence is required to confirm that the zeolite framework remains structurally intact after exposure to and decay of T_2.
5. The study does not examine the effect of common impurities such as H_2O and CO_2, which are known to interact strongly with Ag^+ sites. This omission raises concerns about the material's separation performance and practical applicability in real-world environments.

Reviewer #4

(Remarks to the Author)
The authors present an experimental demonstration of quantum sieving (QS) for the separation of hydrogen isotope mixtures, including tritium, using Ag-exchanged zeolite Y. Separation of hydrogen isotopes via QS is predicted to overcome the limitations of existing technologies due to its high selectivity for tritium. This study is scientifically significant and novel, as it provides the first experimental evidence supporting this potential. However, for publication, the following points should be addressed and clarified:

1) Line 27: The discussion of protium contamination via secondary neutron-induced reactions requires appropriate references to support the statement.
2) Lines 82–84: In the single-gas uptake analysis, it would be helpful to explain why the H_2 uptake is larger than that of D_2 or T_2.
3) Lines 85–86: Was the selectivity of binary mixtures predicted from single-gas TDS analysis calculated and presented? How do these predicted values compare with the experimental results for binary mixtures?
4) Line 96: How was the quantification of heteronuclear isotopologues performed? Although Table S2 provides a brief description in the caption, a reference or additional information regarding the methodology and its accuracy would be beneficial.
5) Table S2: The manuscript states that gas exchange is negligible. However, in the H_2/T_2 gas composition, a significant amount of HT is present relative to H_2, and in the $H_2/D_2/T_2$ data, the amount of HD relative to H_2 is considerable. While it appears that the formation of heteronuclear isotopologues is small compared to the gas equilibrium composition, the authors should carefully justify whether it is appropriate to neglect these species.
6) Line 139: The authors suggest that the structural stability of the Ag-exchanged zeolite is supported by the maintenance of D_2/T_2 gas uptake. However, the uptake varied from 0.22 to 0.26, which may not provide sufficient evidence to conclude that the material possesses radiation stability.

Version 1:

Reviewer comments:

Reviewer #1

(Remarks to the Author)
The authors have fully addressed my concerns. I guess it can be published now.

Reviewer #2

(Remarks to the Author)
The authors have made substantial revisions to the manuscript, and all reviewer concerns have been thoroughly and appropriately addressed. Consequently, the manuscript is acceptable for publication in its current form.

Reviewer #3

(Remarks to the Author)
This revised manuscript presents a compelling and experimentally rigorous validation of Ag-exchanged zeolite Y for the selective separation of hydrogen isotopologues, particularly addressing the long-standing gap in experimental data regarding tritium. The authors have successfully employed TDS to demonstrate exceptional selectivity factors, notably the ST/H value of 244, which significantly outperforms many conventional methods and aligns well with theoretical predictions for chemical affinity quantum sieving. The inclusion of radiation stability tests is a particularly strong addition, effectively mitigating concerns about the radiolytic reduction of Ag(I) sites, a critical factor for practical application in fusion reactor environments. The author has made good revisions to the manuscript and addressed the concerns I raised, and I think that the paper is now ready for publication.
Minor issues:
1. The abstract reports the ternary mixture enrichment ratio as "1:41:175," whereas the Results section specifies the percentages as "0.5%, 18.9%, and 80.6%," suggesting a precision of one decimal place for the percentages. These two representations are not mathematically consistent.
2. The binary selectivity for H2/T2 is reported as "244.1" in the Results, but rounded to "244" in the Abstract and Conclusion.

Reviewer #4

(Remarks to the Author)
The revisions made by the authors are satisfactory and successfully address the previous concerns. In my opinion, the manuscript is now ready for publication.

Open Access This Peer Review File is licensed under a Creative Commons Attribution 4.0 International License, which permits use, sharing, adaptation, distribution and reproduction in any medium or format, as long as you give appropriate credit to the original author(s) and the source, provide a link to the Creative Commons license, and indicate if changes were made.
In cases where reviewers are anonymous, credit should be given to 'Anonymous Referee' and the source.
The images or other third party material in this Peer Review File are included in the article’s Creative Commons license, unless indicated otherwise in a credit line to the material. If material is not included in the article’s Creative Commons license and your intended use is not permitted by statutory regulation or exceeds the permitted use, you will need to obtain permission directly from the copyright holder.
To view a copy of this license, visit https://creativecommons.org/licenses/by/4.0/

NCOMMS-25-85173-T:

Point-by-point response to the Reviewers' comments

We wish to thank all Reviewers for the time and effort they have taken to evaluate our manuscript. We are grateful for their valuable comments leading to substantial improvements throughout the paper.

To substantiate our findings, we conducted extensive additional experiments as well as a recalibration of the mass spectrometer for all hydrogen isotopes. Since the first author left the Helmholtz-Zentrum in Leipzig, we required the support of the new doctoral student in our project, Ms. Jing Liu. Therefore, we acknowledge her essential contribution by including her in the list of authors, i.e., Alexandra Becker, Holger Lippold, **Jing Liu**, Michael Hirscher, Cornelius Fischer.

Reviewer#1 (remarks to the author)

This (communication type) manuscript reports a timely and consequential experimental study of hydrogen isotope separation on Ag(I)-exchanged zeolite Y that, in my view, merits rapid publication after minor revision. The authors present, to my knowledge, the first 'ternary mixture TDS profiles' that directly resolve tritium within both binary and ternary mixtures and extract quantitative selectivities S_D/H=7.7, S_T/H=38.2, S_T/D=2.5 from integrated TDS signals, while documenting minimal isotope exchange products (HD/HT/DT) and stable total uptake upon repeated T_2 exposure, key indicators of data reliability and material robustness under radiolytic conditions. These findings create a needed experimental benchmark for the fusion fuel-cycle community by demonstrating effective chemical-affinity quantum sieving (CAQS) at liquid-nitrogen-accessible temperatures and by reporting ternary $H_2/D_2/T_2$ outcomes that are directly relevant to process design, including a ternary desorption composition with T_2 at ~66-70% depending on whether mixed isotopologues are counted.

There is, however, one non-trivial inconsistency that the authors should resolve before acceptance. In single-gas TDS, the reported absolute uptake for H_2 (~0.72 mmol g^{-1}) is roughly twice that of D_2 (~0.35 mmol g^{-1}) and T_2 (~0.33 mmol g^{-1}) under nominally similar conditions. In most physisorption systems, the number of molecules occupying a given site is comparable across isotopes (or, if anything, slightly higher for the heavier isotope because of lower zero-point energy) so the present inverse trend needs to be explained. A plausible hypothesis is that a calibration constant slips during the initial hydrogen standardization, specifically treating the signal as "H" (atomic) rather than "H_2" (diatomic), which would inflate the apparent H_2 uptake by about a factor of 2. (Indeed, although it is shown to have double the H_2 uptake, the performance of this system will be further enhanced when the calibration constant is corrected.)

Answer: We sincerely thank the Reviewer for acknowledging the significance of this work and for pointing out the discrepancy in the single gas spectra. We have carefully checked our previous calibration runs. The hydrogen signal was correctly assigned to H_2. Seeing the mentioned discrepancy, we decided to repeat the complete calibration process using a fresh $Pd_{98}Ce_2$ alloy sample. Two calibration runs were performed for each isotope, showing good reproducibility:

	1st measurement		2nd measurement	
**Gas**	**Load** (µmol)	**Peak area** (a. u.)	**Load** (µmol)	**Peak area** (a. u.)
H_2	22	9.40	22	9.01
D_2	15	1.59	15	1.52
T_2	13	0.78	13	0.83

This new and consistent calibration results in new constants for each isotope, which have been applied to all TDS data sets (Figs. 1 and 2, Supplementary Figs. 2, 3 and 4). Furthermore, the spectra for the individual isotopes are now in a sound relationship to one another (Supplementary Fig. 2).

The new calibration constants lead to new integrated amounts of the desorbed isotopes and, therefore, to new selectivities for different binary isotopologue mixture experiments, $S_{D/H}$ = 29.2, $S_{T/D}$ = 4.2, and $S_{T/H}$ = 244.1. For the ternary mixture exposure, the resulting percentages are 0.5% for protium, 18.9% for deuterium and 80.6% for tritium. We marked these changes in the revised manuscript.

Changes made:

In manuscript:

Revised:

Fig. 1 (page 10)

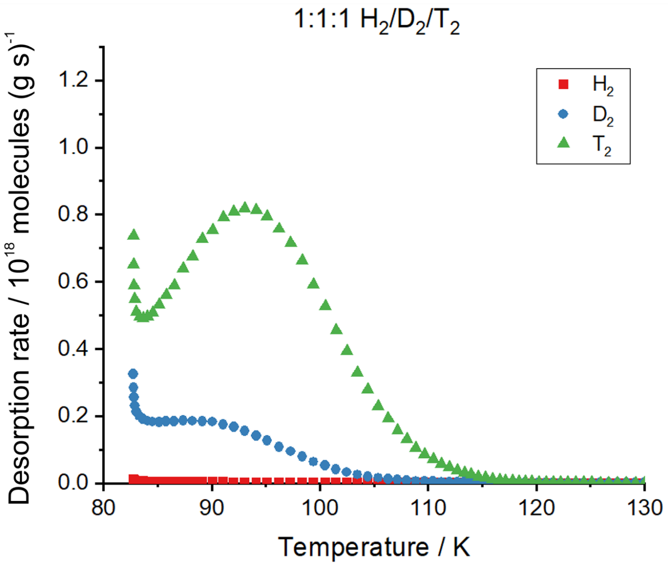

Figure 1. **Desorption spectra of a ternary 1:1:1 hydrogen isotopologue mixture.** Desorption rates for H_2 (red), D_2 (blue) and T_2 (green) during heating at 0.1 K s^{-1} after adsorption on AgY zeolite for 10 min at ~ 83 K. The equimolar mixture was introduced at a total pressure of 1 kPa.

Fig. 2 (page 11)

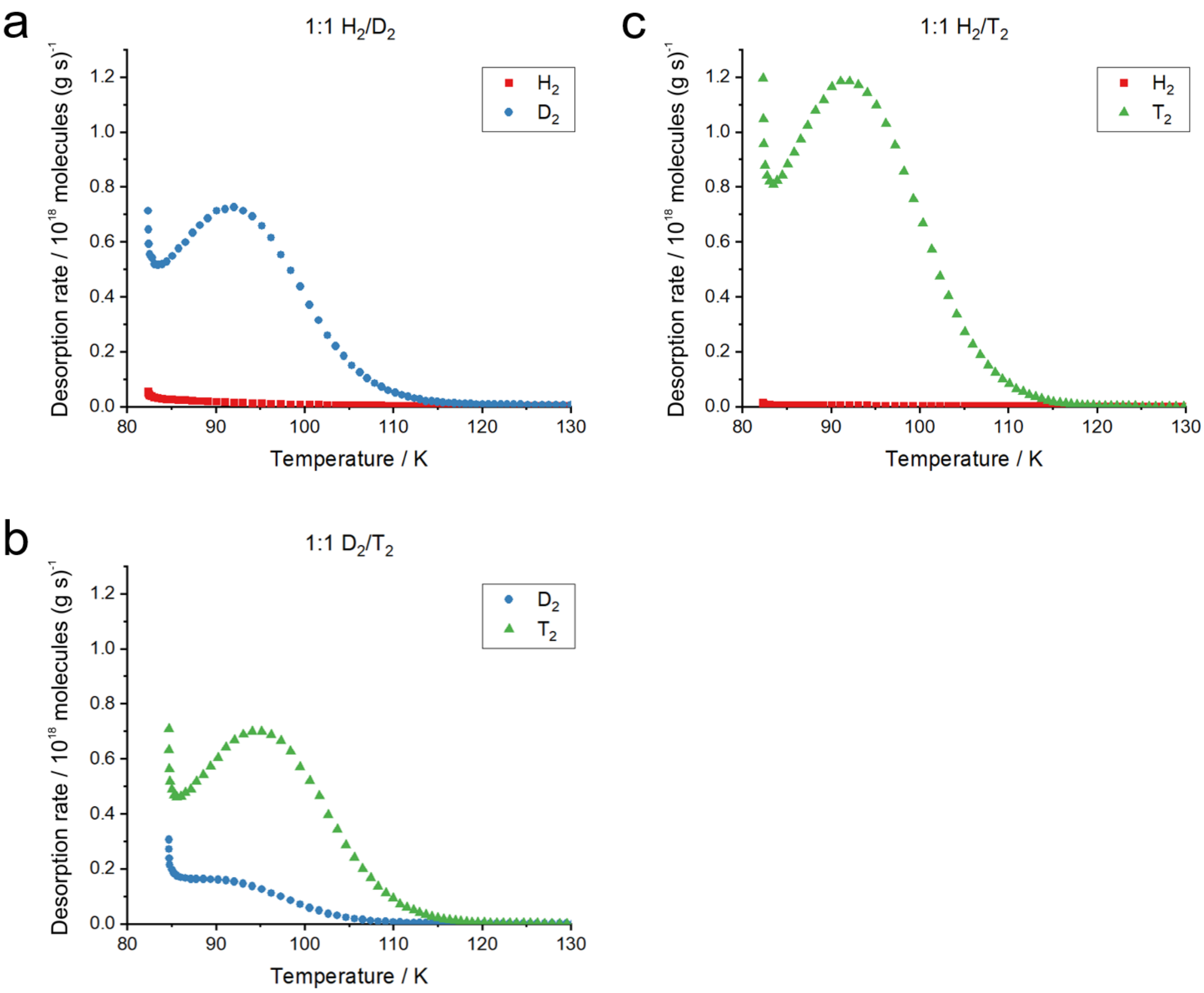

Figure 2. **Desorption spectra of binary 1:1 hydrogen isotopologue mixtures. a** H_2/D_2 (adsorption at ~ 82 K). **b** D_2/T_2 (adsorption at ~ 85 K). **c** H_2/T_2 (adsorption at ~ 82 K). All other conditions correspond to the separation of the ternary system. The equimolar mixtures were introduced at a total pressure of 1 kPa.

All spectra, uptake values in the tables, and selectivities have been adapted to the new calibration constants throughout the supplementary information, too. Former Tables S2 and S3 have been combined in one new Table S2.

In supplementary information:

Revised:

Supplementary Fig. 2 (page 5)

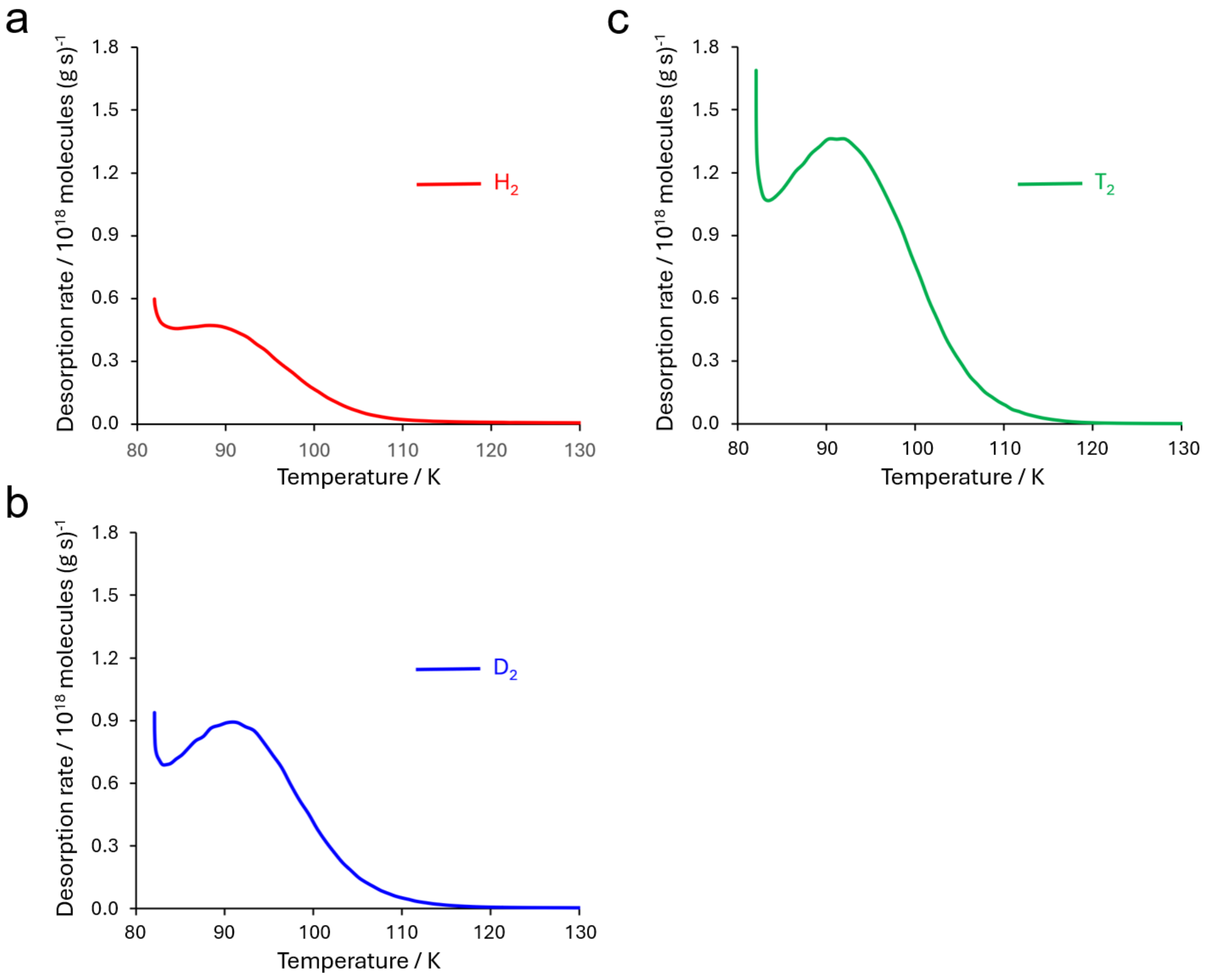

Supplementary Figure 2. **Desorption spectra of the individual hydrogen isotopologues.** Desorption rates during heating at 0.1 K s^{-1} after adsorption on AgY zeolite at ~ 82 K for 10 min. **a** Desorption spectrum of H_2. **b** Desorption spectrum of D_2. **c** Desorption spectrum of T_2. The gases were introduced at a pressure of 1 kPa.

Supplementary Fig. 3 (page 6)
(repetition experiment, not displayed here)

Supplementary Fig. 4 (page 7)
(repetition experiment, not displayed here)

Supplementary Table 1 (page 6)

Supplementary Table 1. **Gas uptakes and rate maximum positions taken from the desorption spectra of the individual hydrogen isotopologues.**

	**1st measurement**		**2nd measurement**	
**Gas**	**Total uptake** (mmol g^{-1})	**Maximum** (K)	**Total uptake** (mmol g^{-1})	**Maximum** (K)
H_2	0.147	88.2	0.145	88.5
D_2	0.260	90.8	0.259	90.3
T_2	0.410	91.1	0.424	90.5

Supplementary Table 2 (page 8)

Supplementary Table 2. **Desorbed amounts of all isotopologues in binary and ternary mixture experiments.**

**System**	**Exposure temperature** (K)	**Amount** (mmol g^{-1})						
		**H_2**	**HD**	**HT**	**D_2**	**DT**	**T_2**	**Total**
H_2/D_2	82.4	0.007	< 0.001	–	0.211	–	–	0.219
H_2/T_2	82.3	0.001	–	0.003	–	–	0.344	0.348
D_2/T_2 (1)	84.7	–	–	–	0.048	< 0.007	0.201	0.256
D_2/T_2 (2)	83.2	–	–	–	0.066	< 0.006	0.219	0.291
$H_2/D_2/T_2$	82.8	0.001	< 0.002	unknown	0.055	< 0.004	0.236	0.298

Reviewer#2 (remarks to the author)

Becker and coworkers have reported in this manuscript the separation of T_2 from hydrogen isotopes utilizing Ag-exchanged zeolite Y at temperatures exceeding 77 K. This achievement represents a significant and exciting advancement in the field of hydrogen isotope separation. The manuscript will be deemed appropriate for publication in Nature Communications provided that the following concerns are adequately addressed.

We thank the Reviewer for the positive evaluation of our work regarding its scientific value. We appreciate the Reviewer's insightful comments, leading to essential amendments of the paper by additional studies and important information that was in fact missing.

Question 1: The AgY zeolite was synthesized through ion exchange from NaY using $AgNO_3$, as detailed in the manuscript. What is the Ag content in the target AgY? Additionally, will the Ag content influence the T_2 separation performance?

Answer: After treating the NaY zeolite three times with an excess of $AgNO_3$, Na was found to be fully exchanged by Ag, based on elemental analysis by ICP-OES. The Ag/Al ratio was determined to be 1.04, indicating that all Al positions are accompanied by Ag. The residual Na content was below the detection limit of 2.6 ng/g.

Partial hydrogen adsorption on sites other than Ag, lacking the big difference in the zero-point energies of bound $H_2/D_2/T_2$, would indeed imply a considerable loss of selectivity. However, in a previous study, we found that there is hardly any H adsorption on a "bare" zeolite at temperatures higher than 75 K (DOI: 10.1021/acs.inorgchem.2c00028). Thus, incomplete exchange will not significantly alter the T_2 separation performance in terms of selectivity, but certainly in terms of yield due to the lower adsorption capacity.

Changes made:

In manuscript:

Added:

(page 5, Methods)

"Elemental analysis by ICP-OES indicated complete replacement of Na by Ag.[(MS Ref. 16)]"

In supplementary information:

Added:

(page 3)

"For ion exchange, 1 g of zeolite NaY (Clariant, Germany) was treated in 125 mL of a 0.1 M $AgNO_3$ solution (2.12 g of $AgNO_3$ dissolved in 125 mL of deionized argon-saturated water) for 2 h under an Ar atmosphere in a vessel protected from light. The material was then separated by vacuum filtration and was washed with deionized argon-saturated water. The procedure was repeated another two times. The final product was dried at 80°C for 12 h and was stored in the absence of light.

The elemental composition was determined by ICP-OES after digestion. The Ag/Al ratio was found to be 1.04, indicating that Na was completely exchanged for Ag, occupying all Al sites. The residual Na content was below the detection limit of 2.6 ng g^{-1}. Previous studies by XRD have shown that the zeolite Y structure is well preserved after ion exchange. The water content was 15.1 wt.% as determined by TG/DSC.[SI Ref. 1]"

<u>Question 2:</u> T_2 is a radioactive substance that may potentially damage the structure of the sorbent as mentioned in the manuscript. A comparative analysis of the structural integrity of AgY zeolite before and after exposure to T_2 should be conducted, focusing on PXRD, physisorption, and Ag content.

<u>Answer:</u> We thank the Reviewer for raising this point. We fully agree that potential effects of radiation must be thoroughly considered in connection with separation studies including tritium. Regarding the integrity of the lattice structure, amorphization is observed at electron doses many orders of magnitude higher than applied during short-term T_2 exposure at low pressure (DOI: 10.1016/j.cossms.2005.04.002). PXRD would require larger amounts of (contaminated) zeolite to be handled at an external facility, which is an issue in respect of safety regulations and unlikely to yield indications of structural alterations, given that the electron energy is very low (max. 18.6 keV).

As stated by the Reviewer, it is appropriate to focus on the binding sites themselves, in the light of the fact that Ag(I) is reduced in consequence of ionizing radiation, a process that is widely used in photo emulsions for dosimetry and autoradiography. To examine whether the Ag(I) sites are affected by the beta radiation, we exposed the zeolite to a T_2 atmosphere at 82 K for a time period considerably exceeding the contact time in the separation process, and used TDS records for D_2 as an indicator of changes in the Ag(I) content, based on triplicate measurements before and after T_2 contact. There was no significant change neither in the adsorbed amounts nor in the desorption profiles. With these additional measurements, we are now able to substantiate on a statistically sound basis that the Ag(I) sites are unaffected by beta radiation under separation conditions.

<u>Changes made:</u>

In manuscript:

Added:

(page 4)

"In view of the well-known sensitivity of Ag(I) ions to reduction in consequence of ionizing radiation (used in photo emulsions for dosimetry and autoradiography), the question needs to be addressed as to whether the Ag(I) adsorption sites are affected by the beta radiation. After 4 hours of exposure to T_2 under the conditions applied in the separation experiments (1 kPa T_2, 82 K), no significant change in the desorption spectra was detected based on triplicate TDS records for D_2 before and after T_2 contact (Supplementary Fig. 5, Supplementary Table 3). Obviously, the extent of ionization within the zeolite lattice is too low for an electron transfer to the Ag(I) ions. Multiple exposure to T_2-containing gas mixtures will not affect the separation performance."

In supplementary information:

Added:

Supplementary Fig. 5 (page 9)

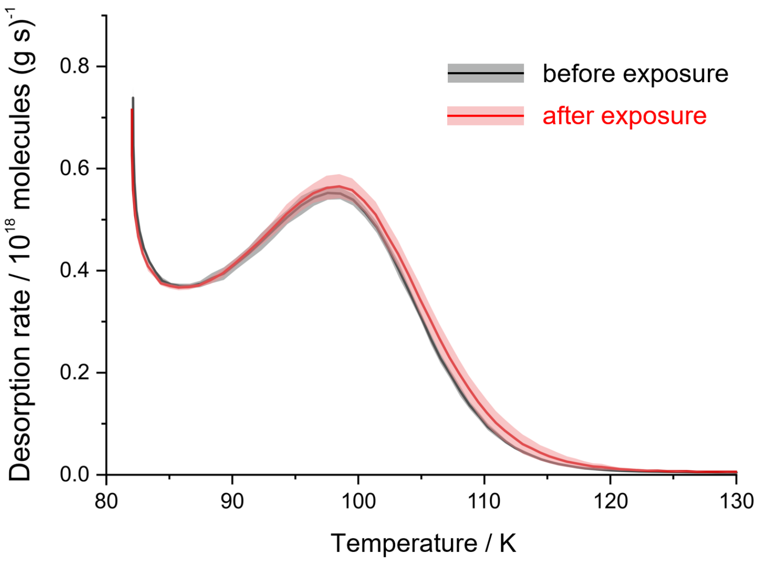

Supplementary Figure 5. **Desorption spectra of D_2 before and after prolonged exposure of Ag(I)-exchanged zeolite to T_2.** Desorption rates during heating at 0.1 K s^{-1} after adsorption at ~ 82 K for 10 min. D_2 was introduced at a pressure of 1 kPa. The lines represent the mean values, the shaded areas show the standard deviations for triplicate TDS records before and after T_2 exposure of the zeolite. For exposure, T_2 was introduced at a pressure of 1 kPa and was left in the adsorption equilibrium at ~ 82 K for 4 h. T_2 was then desorbed in a TDS run. The total number of decays of adsorbed T_2 during exposure was 1.22×10^{16}.

Supplementary Table 3 (page 9)

Supplementary Table 3. **Total uptakes of D_2 on Ag(I)-exchanged zeolite before and after prolonged exposure to T_2.**

**No. of TDS run**	**Gas uptake** (mmol g^{-1})	
	**before exposure**	**after exposure**
1	0.191	0.212
2	0.195	0.194
3	0.199	0.201
**Mean value:**	0.195	0.202
**Standard deviation:**	0.004	0.009

Question 3: Measurements of single-component physisorption for H_2, D_2, and T_2 at temperatures ranging from 80 to 85 K are required.

Answer:

Physisorption on the zeolite's skeleton, as opposed to Ag(I) sites adsorption, does not occur at temperatures higher than 75 K. This has been shown in a previous study using the same material under the same conditions (DOI: 10.1021/acs.inorgchem.2c00028), now indicated in the manuscript. Based on these results, the residual evacuation tailing (initial part of the spectra) is constituted by gas being desorbed from the Ag(I) sites. We include this tailing in the integration rather than using the minimum as the lower limit, since this would lead to an underestimation of adsorbed amounts. The source of the given uptakes is now specified, as this information was indeed missing.

Changes made:

In manuscript:

Added:

(page 3)

"Physisorption on sites other than Ag does not occur above liquid-nitrogen temperature.[MS Ref. 16]"

Added:

(page 6, Methods)

"The integrals of the desorption spectra, corresponding to the adsorbed amounts, were calculated based on the entire temperature range."

In supplementary information:

Added:

(page 6)

"The uptakes were determined by integrating the desorption spectra starting from the exposure temperature."

<u>Question 4:</u> The complete name of 'ITER' should be presented upon its initial mention in the main text.

<u>Answer:</u> The full designation has been included.

<u>Changes made:</u>

In manuscript:

Added:

(page 1)

"the International Thermonuclear Experimental Reactor (ITER)"

<u>Question 5:</u> The Ref. 4 is incorrectly formatted, and the page number for Ref. 9 is also incorrect.

<u>Answer:</u> We apologize for these mistakes, and we thank the Reviewer for the very careful reading. We have reformatted the reference list according to the journal guidelines, making sure that all numbers are correct. We have replaced Ref. 4 by a more suitable citation.

<u>Changes made:</u>

In manuscript:

Revised:

Ref. 4 "Kocher, R., Auer, C., Flynn, M., Kocher, R. & Rothstein, L. Bulletins. *Bulletin of the Atomic Scientists* **59**, 6-11 (2003)"
replaced by "Kim, K., Kim, K., Son, S. H., Kim, W.-S. Manufacturing process of self-luminous glass tube (SLGT) utilizing tritium gas: Design of tritium handling facilities. *Korean J. Chem. Eng.* **21**, 562-566 (2004)"

Reviewer#3 (remarks to the author)

This manuscript describes the separation of $H_2/D_2/T_2$ mixtures using Ag(I)-exchanged zeolite Y, as evaluated by TDS. While the handling of tritium in the experiments is technically challenging and commendable, the study offers limited novelty and fails to provide significant beyond the existing literature. This is particularly evident in light of the comprehensive study by Xiong et al, which has already elucidated the mechanism and selectivity trends for T_2/D_2 separation in Ag^+-exchanged zeolites. Consequently, the work in its current form is not suitable for publication in Nature Communications. The specific concerns are outlined below.

We sincerely appreciate the Reviewer's acknowledgement of the challenges to be tackled for including tritium in the exploration of isotope separation processes, and we value the thoughtful feedback, which has led to important revisions of the manuscript. We will strive to better highlight the significance of our work in the following.

The prominent study by Xiong et al. was a key motivation for our work. We referred to it accordingly. Their DFT-based predictions of selectivities for T-containing isotopologue mixtures have literally "awaited" experimental confirmation – a task that had previously been circumvented for solid reasons, but has now been successfully addressed.

Question 1: The authors must clarify the new fundamental insights or performance advantages offered by this work relative to prior studies. The feasibility, mechanism, and selectivity of Ag^+-exchanged zeolites for separating D_2/H_2 and T_2/D_2 have already been established in the literature (Chemical Engineering Journal, 2024, 493, 152673). Simply extending the application from binary to ternary gas mixtures is considered insufficient to constitute a significant advance.

Answer: The scientific value of the paper by Song et al. is undisputed, and has been cited appropriately. While H_2/D_2 separation was comprehensively investigated based on full TDS records, T_2/D_2 selectivities were determined in a single-point approach by offline analysis of gas aliquots sampled after equilibration and after desorption, yielding a cumulative result.

Applying different experimental procedures for subsystems within an ensemble always bears the risk of inconsistencies. For a sound intercomparison, the same kind of information is needed for all isotopes and for all isotope mixtures. With our study, we provide a complete data set of TDS spectra after exposure to single, binary and ternary gaseous isotopes. Therefore, we do not regard these first TDS-based separation of a $H_2/D_2/T_2$ system as a sheer extension of the previous, very limited information gained by a single-point gas analysis.

The gain in information obtained from temperature-resolved desorption rate spectra is certainly beyond debate. Our measurements after exposure to different isotope mixtures, including ternary, go far beyond any previous study and give new fundamental insights, e.g., on isotope specific desorption rate maxima, which are related to the respective binding energies. This is now explicated more specifically in the paper.

Our comprehensive TDS study of all binary and ternary systems additionally includes an analysis of mixed isotopologues formed under separation conditions, which is crucial because isotope exchange can be significant in the adsorbed state, disqualifying the separation strategy. Another important aspect, as pointed out by the Reviewer in Question 4, is the radiation resilience of the material. Both aspects are explicitly considered in our paper. To address the

question of resistance to radiation on a reliable basis, additional experiments have been included in the revised version.

Changes made:

In manuscript:

Added:

(page 4)

"The higher desorption temperatures of the heavier isotopologues confirm their higher adsorption enthalpies, which correlates with their preferential adsorption by CAQS. Moreover, this difference in adsorption energies explains why the uptake ratios in isotopologue mixtures are more pronounced than in the corresponding single-gas systems (Supplementary Fig. 2). During gas exposure, the exchange of isotopologues at adsorption sites initially occupied by lighter isotopes results in uptake ratios that differ from those inferred from single-gas experiments."

Question 2: The discussion of the CAQS mechanism remains purely qualitative. To substantiate these claims, it is essential to provide quantitative support, such as through relevant theoretical calculations (e.g., adsorption energies, binding strengths).

Answer: Here we would like to refer to a previous paper where detailed DFT calculations have been performed for H_2/D_2 adsorption on the AgY zeolite under study (DOI: 10.1021/acs.inorgchem.2c00028). We demonstrated that D_2/H_2 selectivity is governed by the respective changes in the zero-point energies upon adsorption, spread by the strong interaction with Ag as a binding site. We think that the validity of this mechanism can be extended to the third isotope without separate DFT evidence, as this would go beyond the scope of this communication. We gladly take it as a motivation for a future publication.

The data originally provided for the single-gas systems were indeed not in line with the CAQS mechanism based on zero-point energies, which should be reflected by an increase in the adsorbed amounts in the order $H_2<D_2<T_2$. Having recalibrated the MS, the adsorbed amounts for the single isotopologues are now consistent in their relationship (Supplementary Table 1).

Changes made:

In manuscript:

Added:

(page 3)

"This allows for a direct comparison with previous studies on protium-deuterium mixtures, where the validity of the CAQS mechanism at strong Ag sites has been demonstrated by DFT calculations.[MS Ref. 16]"

In supplementary information:

Revised:

Supplementary Table 1 (page 6)

Supplementary Table 1. **Gas uptakes and rate maximum positions taken from the desorption spectra of the individual hydrogen isotopologues.**

	1st measurement		2nd measurement	
**Gas**	**Total uptake** (mmol g^{-1})	**Maximum** (K)	**Total uptake** (mmol g^{-1})	**Maximum** (K)
H_2	0.147	88.2	0.145	88.5
D_2	0.260	90.8	0.259	90.3
T_2	0.410	91.1	0.424	90.5

Question 3: The manuscript omits key material properties, including Ag^+ loading, cation exchange degree, and site distribution. Given the direct influence of Ag^+ concentration on adsorption enthalpy and isotopic selectivity, this gap critically undermines both the reproducibility of the work and the interpretation of the underlying mechanism.

Answer: This information was indeed missing, as was also pointed out by Reviewer#2. ICP-OES analysis yielded an Ag/Al ratio of 1.04 while the Na content was below the detection limit, indicating complete exchange of Na for Ag and regular site distribution at the Al positions. Xiong et al. successfully predicted selectivities by DFT assuming one type of binding site. The influence on selectivity is less critical because adsorption is restricted to the Ag sites at temperatures higher than 75 K (DOI: 10.1021/acs.inorgchem.2c00028).

Changes made:

In manuscript:

Added:

(page 5, Methods)

"Elemental analysis by ICP-OES indicated complete replacement of Na by Ag.[MS Ref. 16]"

In supplementary information:

Added:

(page 3)

"For ion exchange, 1 g of zeolite NaY (Clariant, Germany) was treated in 125 mL of a 0.1 M $AgNO_3$ solution (2.12 g of $AgNO_3$ dissolved in 125 mL of deionized argon-saturated water) for 2 h under an Ar atmosphere in a vessel protected from light. The material was then separated by vacuum filtration and was washed with deionized argon-saturated water. The procedure was

repeated another two times. The final product was dried at 80°C for 12 h and was stored in the absence of light.

The elemental composition was determined by ICP-OES after digestion. The Ag/Al ratio was found to be 1.04, indicating that Na was completely exchanged for Ag, occupying all Al sites. The residual Na content was below the detection limit of 2.6 ng g^{-1}. Previous studies by XRD have shown that the zeolite Y structure is well preserved after ion exchange. The water content was 15.1 wt.% as determined by TG/DSC.[SI Ref. 1]"

Question 4: The assertion that the material exhibits radiation stability is not backed by structural characterization data (e.g., XRD, BET, EXAFS). Direct evidence is required to confirm that the zeolite framework remains structurally intact after exposure to and decay of T_2.

Answer: This question, also raised by Reviewer#2, is in fact critical. Including tritium in separation studies not only requires technical solutions for radiation protection but also careful consideration of how the radiation affects the material under investigation. Damages of the framework can be excluded in consideration of the very low beta energy of max. 18.6 keV. Structural changes are observed at doses exceeding the applied doses by many orders of magnitude (DOI: 10.1016/j.cossms.2005.04.002). A "quick check" in external laboratories equipped with XRD, BET or EXAFS is not possible for lack of the regulatory and technical prerequisites to handle tritium. (Some contamination is still present after desorption, and the required sample amounts are rather high.)

The actual point of weakness is the silver, considering that Ag(I)-containing photo emulsions are widely used for detecting radiation. Reduction might also take place on the AgY zeolite. To investigate this, the material was exposed to a T_2 atmosphere at 82 K for a duration significantly longer than the contact time during separation. Changes in the Ag(I) content were then evaluated using TDS measurements of D_2, based on triplicate datasets collected before and after T_2 exposure. Neither the adsorption capacities nor the desorption profiles showed any statistically significant variation. These results provide robust evidence that, under separation conditions, the Ag(I) sites remain unaffected by beta radiation.

Changes made:

In manuscript:

Added:

(page 4)

"In view of the well-known sensitivity of Ag(I) ions to reduction in consequence of ionizing radiation (used in photo emulsions for dosimetry and autoradiography), the question needs to be addressed as to whether the Ag(I) adsorption sites are affected by the beta radiation. After 4 hours of exposure to T_2 under the conditions applied in the separation experiments (1 kPa T_2, 82 K), no significant change in the desorption spectra was detected based on triplicate TDS records for D_2 before and after T_2 contact (Supplementary Fig. 5, Supplementary Table 3). Obviously, the extent of ionization within the zeolite lattice is too low for an electron transfer to the Ag(I) ions. Multiple exposure to T_2-containing gas mixtures will not affect the separation performance."

In supplementary information:

Added:

Supplementary Fig. 5 (page 9)

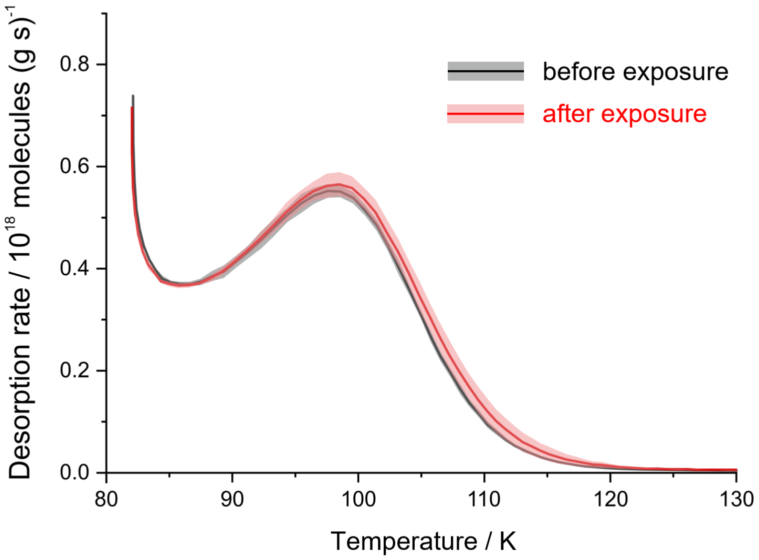

Supplementary Figure 5. **Desorption spectra of D_2 before and after prolonged exposure of Ag(I)-exchanged zeolite to T_2.** Desorption rates during heating at 0.1 K s^{-1} after adsorption at ~ 82 K for 10 min. D_2 was introduced at a pressure of 1 kPa. The lines represent the mean values, the shaded areas show the standard deviations for triplicate TDS records before and after T_2 exposure of the zeolite. For exposure, T_2 was introduced at a pressure of 1 kPa and was left in the adsorption equilibrium at ~ 82 K for 4 h. T_2 was then desorbed in a TDS run. The total number of decays of adsorbed T_2 during exposure was 1.22×10^{16}.

Supplementary Table 3 (page 9)

Supplementary Table 3. **Total uptakes of D_2 on Ag(I)-exchanged zeolite before and after prolonged exposure to T_2.**

**No. of TDS run**	**Gas uptake** (mmol g^{-1})	
	**before exposure**	**after exposure**
1	0.191	0.212
2	0.195	0.194
3	0.199	0.201
**Mean value:**	0.195	0.202
**Standard deviation:**	0.004	0.009

<u>Question 5:</u> The study does not examine the effect of common impurities such as H_2O and CO_2, which are known to interact strongly with Ag^+ sites. This omission raises concerns about the material's separation performance and practical applicability in real-world environments.

It would in fact be interesting to include (at least) CO_2 in our separation studies to evaluate its effect. For the present work, we made sure that any adsorbed impurities were completely removed prior to the TDS experiments, heating the zeolite two times at 500 K for 2 hours while monitoring the masses 18 and 44. Such activation is also required in any potential application. The purities of used H_2 and D_2 gases are now specified in the Methods section. The tritium source is kept under vacuum and is baked out prior to use to separate the decay product ^3He.

Regarding real-world environments (albeit nuclear fusion is not yet part of the real world), CO_2 is not involved in the fuel cycle (DOI: 10.1134/S1063778815100026). HTO may occur and must be separated before treating $H_2/D_2/T_2$.

<u>Changes made:</u>

In manuscript:

Added:

(page 5, Methods)

"H_2 with a purity of 99.9992% was supplied by Air Products (Germany), D_2 with a purity of 99.8% was purchased from Air Liquide (Germany)."

Reviewer #4 (remarks to the author)

The authors present an experimental demonstration of quantum sieving (QS) for the separation of hydrogen isotope mixtures, including tritium, using Ag-exchanged zeolite Y. Separation of hydrogen isotopes via QS is predicted to overcome the limitations of existing technologies due to its high selectivity for tritium. This study is scientifically significant and novel, as it provides the first experimental evidence supporting this potential. However, for publication, the following points should be addressed and clarified.

We wish to thank the Reviewer for the positive assessment of the scientific merit of our work, and we appreciate the helpful comments pointing out inconsistencies that were in strong need of rectification.

Question 1: Line 27: The discussion of protium contamination via secondary neutron-induced reactions requires appropriate references to support the statement.

Answer: We have replaced the cited articles by papers specifically focused on (n,p) reactions generating protium in fusion reactors.

Changes made:

In manuscript:

Revised:

Ref. 2 "Neugebauer, C., Hörstensmeyer, Y. & Day, C. Technology development for isotope rebalancing and protium removal in the EU-DEMO fuel cycle. *Fusion Sci. Technol.* **76**, 215-220 (2020)"
Ref. 3 "Narcisi, V., Sansosti, L. & Santucci, A. Experimental assessment of palladium-silver membrane for isotope separation, rebalancing and protium removal. *Fusion Eng. Des.* **221**, 115406 (2025)"
replaced by
Ref. 2 "Tel, E. Study on some structural fusion materials for (*n*,*p*) reactions up to 30 MeV Energy. *J. Fusion Energ.* **29**, 332-336 (2010)"
Ref. 3 "Parashari, S., Mukherjee, S., Vansola, V., Makwana, R., Singh, N. L. & Pandey, B. Investigation of (n, p), (n, 2n) reaction cross sections for Sn isotopes for fusion reactor applications. *Appl. Radiat. Isot.* **133**, 31-37 (2018)"

Question 2: Lines 82–84: In the single-gas uptake analysis, it would be helpful to explain why the H_2 uptake is larger than that of D_2 or T_2.

Answer: We thank the Reviewer for highlighting this inconsistency, also noted by Reviewer#1. In response, we have recalibrated the MS. All TDS data sets (Figs. 1 and 2, Supplementary Figs. 2, 3, and 4) have been revised accordingly, resolving the discrepancies in the spectral data for the individual isotopologues.

The revisions made in response to Reviewer#1 are reproduced here for convenience.

Changes made:

In manuscript:

Revised:

Fig. 1 (page 10)

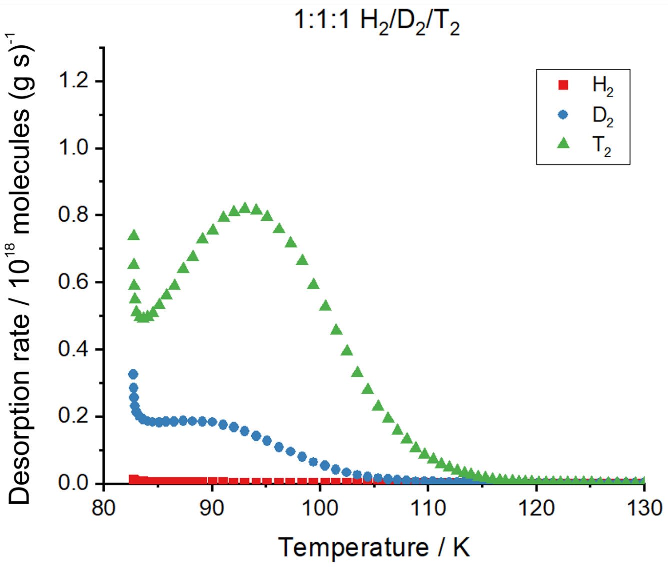

Figure 1. **Desorption spectra of a ternary 1:1:1 hydrogen isotopologue mixture.** Desorption rates for H_2 (red), D_2 (blue) and T_2 (green) during heating at 0.1 K s^{-1} after adsorption on AgY zeolite for 10 min at ~ 83 K. The equimolar mixture was introduced at a total pressure of 1 kPa.

Fig. 2 (page 11)

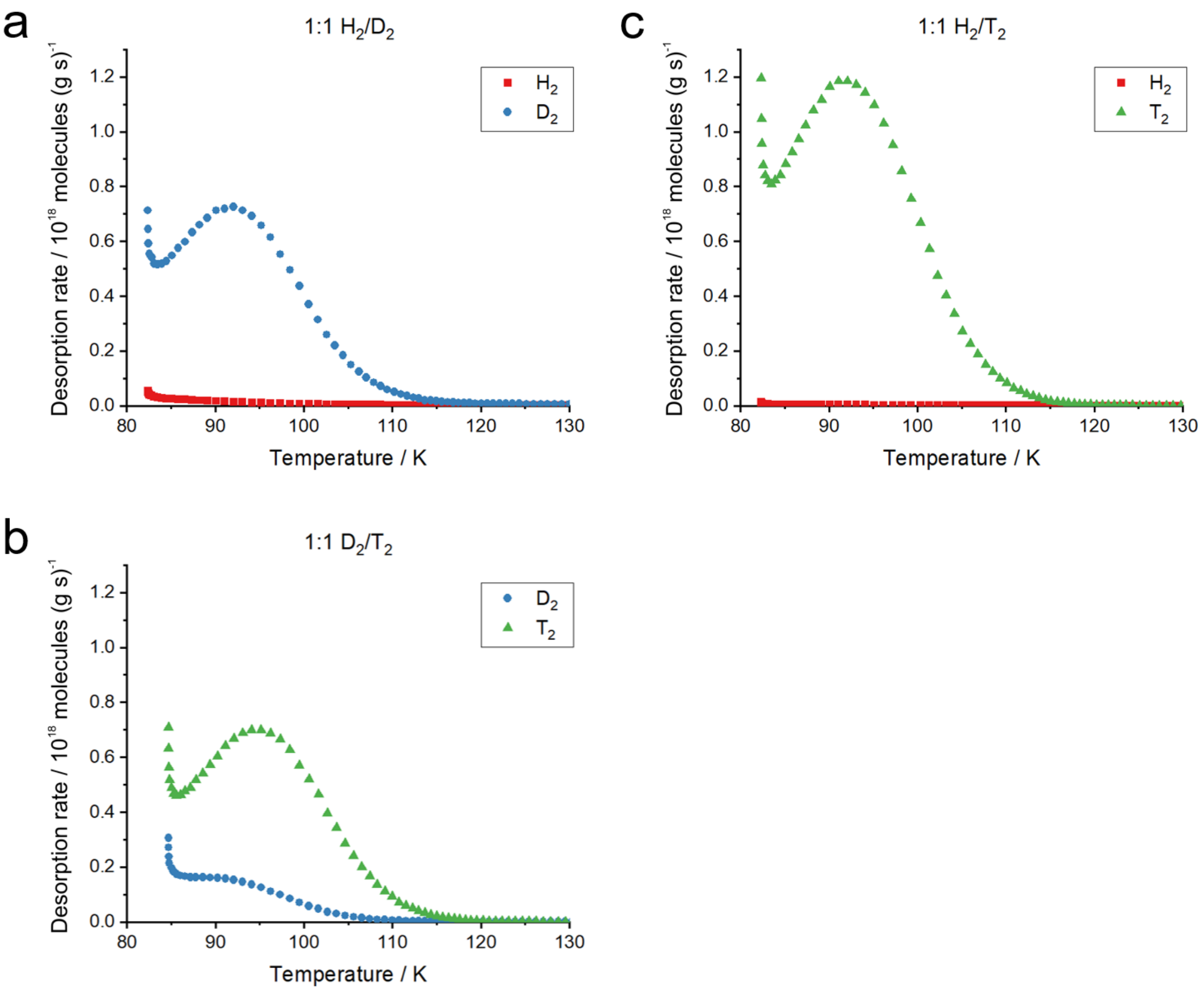

Figure 2. **Desorption spectra of binary 1:1 hydrogen isotopologue mixtures. a** H_2/D_2 (adsorption at ~ 82 K). **b** D_2/T_2 (adsorption at ~ 85 K). **c** H_2/T_2 (adsorption at ~ 82 K). All other conditions correspond to the separation of the ternary system. The equimolar mixtures were introduced at a total pressure of 1 kPa.

All indications of uptakes and selectivities have been adapted throughout the manuscript.

In supplementary information:

Revised:

Supplementary Fig. 2 (page 5)

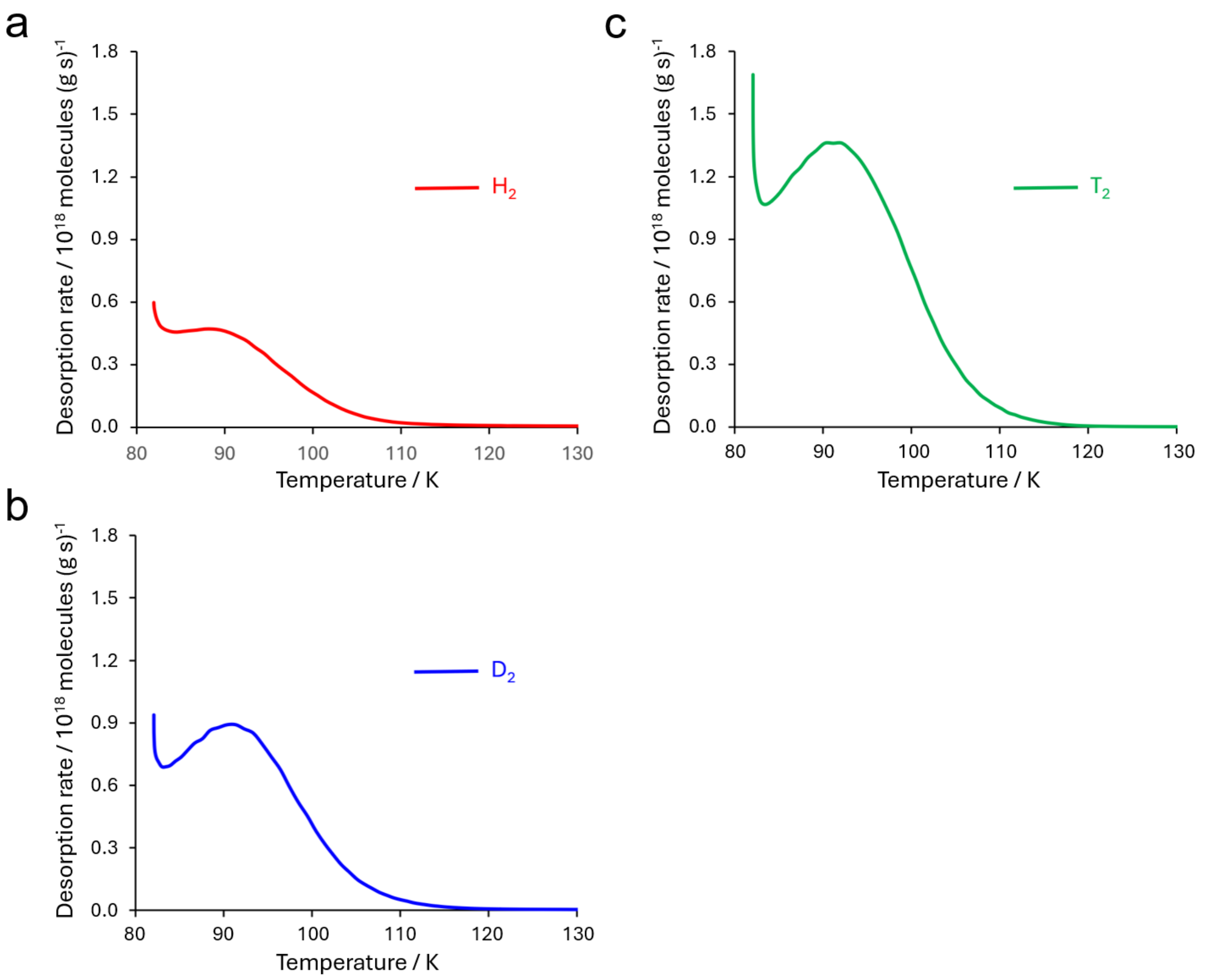

Supplementary Figure 2. **Desorption spectra of the individual hydrogen isotopologues.** Desorption rates during heating at 0.1 K s^{-1} after adsorption on AgY zeolite at ~ 82 K for 10 min. **a** Desorption spectrum of H_2. **b** Desorption spectrum of D_2. **c** Desorption spectrum of T_2. The gases were introduced at a pressure of 1 kPa.

Supplementary Fig. 3 (page 6)
(repetition experiment, not displayed here)

Supplementary Fig. 4 (page 7)
(repetition experiment, not displayed here)

Supplementary Table 1 (page 6)

Supplementary Table 1. **Gas uptakes and rate maximum positions taken from the desorption spectra of the individual hydrogen isotopologues.**

	1st measurement		2nd measurement	
**Gas**	**Total uptake** (mmol g^{-1})	**Maximum** (K)	**Total uptake** (mmol g^{-1})	**Maximum** (K)
H_2	0.147	88.2	0.145	88.5
D_2	0.260	90.8	0.259	90.3
T_2	0.410	91.1	0.424	90.5

Supplementary Table 2 (page 8)

Supplementary Table 2. **Desorbed amounts of all isotopologues in binary and ternary mixtures.**

**System**	**Exposure temperature** (K)	**Amount** (mmol g^{-1})						
		**H_2**	**HD**	**HT**	**D_2**	**DT**	**T_2**	**Total**
H_2/D_2	82.4	0.007	< 0.001	–	0.211	–	–	0.219
H_2/T_2	82.3	0.001	–	0.003	–	–	0.344	0.348
D_2/T_2 (1)	84.7	–	–	–	0.048	< 0.007	0.201	0.256
D_2/T_2 (2)	83.2	–	–	–	0.066	< 0.006	0.219	0.291
$H_2/D_2/T_2$	82.8	0.001	< 0.002	unknown	0.055	< 0.004	0.236	0.298

Question 3: Lines 85–86: Was the selectivity of binary mixtures predicted from single-gas TDS analysis calculated and presented? How do these predicted values compare with the experimental results for binary mixtures?

Answer: Our phrasing "The selectivities for the binary mixtures were calculated from the individual gas uptakes…" was misleading. We did not attempt to predict selectivities in mixtures based on the single-gas TDS data, given that the preference for the heavier isotopes is strongly overexpressed in the mixtures (comparing, e.g., Fig. 1 with Supplementary Fig. 2). This effect is not unusual, here best explained by mass-dependent hindrance of desorption during the equilibration process. This is now pointed out in the text.

Changes made:

In manuscript:

Added:

(page 4)

"The higher temperatures required for the maximum desorption rates of the heavier isotopologues confirm their higher adsorption energies. This difference in adsorption energies

also explains why the uptake ratios are more pronounced in mixtures than in the corresponding single-gas systems (Supplementary Fig. 2). During gas exposure, the exchange of isotopologues at adsorption sites initially occupied by lighter isotopes results in uptake ratios that differ from those inferred from single-gas experiments.”

Question 4: Line 96: How was the quantification of heteronuclear isotopologues performed? Although Table S2 provides a brief description in the caption, a reference or additional information regarding the methodology and its accuracy would be beneficial.

Answer: Using the mean values of the calibration factors of the neighboring homonuclear isotopologues was possible without concern because the determined factors were on a straight line. In addition, however, it had to be considered that the resolution of commercial QMSs is limited for trace amounts of low *m/z* gases due to the asymmetric peak tailing, reaching out from strong signals in the adjacent *m/z*+1 channel. This makes a quantitative measurement of HD and DT formation in hydrogen isotope mixtures difficult. Therefore, we have carefully measured the influence of peak tailing on *m/z* = 3 for pure D_2 and on *m/z* = 5 for pure T_2 as a function of peak intensities and derived a lower bound of the tailing contributions to be subtracted, allowing for a conservative estimate on the contribution of isotope exchange. (The signal for HT in H_2/T_2 systems is unaffected.) Supplementary Table 2 and the belonging explanations have been amended accordingly.

Changes made:

In supplementary information:

Added:

(page 8)

“Desorbed amounts of HD, HT and DT were calculated from the MS data using calibration factors derived by averaging the calibration factors of the neighboring homonuclear isotopologues, based on a linear correlation. For HD and DT, an overlap by asymmetric peak tailing extending from *m/z* = 4 and *m/z* = 6, respectively, had to be taken into account. For a conservative estimate on the maximum contributions of isotope exchange, a lower bound of the tailing contributions was derived from data analyses for single gas systems at varying D_2 and T_2 peak intensities.”

Question 5: Table S2: The manuscript states that gas exchange is negligible. However, in the H_2/T_2 gas composition, a significant amount of HT is present relative to H_2, and in the $H_2/D_2/T_2$ data, the amount of HD relative to H_2 is considerable. While it appears that the formation of heteronuclear isotopologues is small compared to the gas equilibrium composition, the authors should carefully justify whether it is appropriate to neglect these species.

Answer: We fully consent to this objection. The extent of isotope exchange is certainly small if we refer to the applied gas mixture, however, in the adsorbed phase, T_2 is highly enriched, and the H_2 fraction is small. Due to this strongly asymmetric composition of the adsorbed phase, the inferior component is involved at a proportion that cannot be termed as negligible. In fact, a relatively high fraction of adsorbed H_2 molecules is affected in the H_2/T_2 system. While this is not a methodical drawback since the separation strategy aims at the enrichment and isolation

of the preferred component without any necessity of regaining the small amount of (partly spoiled) H_2, the usage of terms like “minimal” and “negligible” was in need of adjustment. The paragraph has been rewritten.

Changes made:

In manuscript:

Revised:

(page 4)

“For all gas mixtures, formation of heteronuclear isotopologues (HD, HT, DT) by isotope exchange was found to be very minor compared to the total adsorbed amounts (Supplementary Table 2). Owing to the strong enrichment of the heaviest isotope in the adsorbed phase, the small fraction of adsorbed H_2 undergoes an enhanced isotope scrambling. Still, the resulting heteronuclear isotopologues are negligible in relation to the total amount of H_2 involved in the separation process. The adsorbate compositions suggest that isotope exchange is largely confined to the adsorbed state.”

“Furthermore, a negligible isotope exchange is observed relative to the total quantity of exposed gas, and the AgY zeolite proves to be radiation-resistant,”

Question 6: Line 139: The authors suggest that the structural stability of the Ag-exchanged zeolite is supported by the maintenance of D_2/T_2 gas uptake. However, the uptake varied from 0.22 to 0.26, which may not provide sufficient evidence to conclude that the material possesses radiation stability.

Answer: The Reviewer is absolutely correct in his objection that the statistical basis provided was not sufficient to substantiate our conclusion. Therefore, we have conducted additional experiments, exposing the AgY zeolite to T_2 for a much longer time period under separation conditions, i.e., at low temperature since potential effects were expected to be most pronounced in the adsorbed state. Triplicate TDS runs with D_2 before and after exposure were performed to provide reliable statistics. No changes were observed within the limits of accuracy.

Changes made:

In manuscript:

Added:

(page 4)

“In view of the well-known sensitivity of Ag(I) ions to reduction in consequence of ionizing radiation (used in photo emulsions for dosimetry and autoradiography), the question needs to be addressed as to whether the Ag(I) adsorption sites are affected by the beta radiation. After 4 hours of exposure to T_2 under the conditions applied in the separation experiments (1 kPa T_2, 82 K), no significant change in the desorption spectra was detected based on triplicate TDS records for D_2 before and after T_2 contact (Supplementary Fig. 5, Supplementary Table 3).

Obviously, the extent of ionization within the zeolite lattice is too low for an electron transfer to the Ag(I) ions. Multiple exposure to T_2-containing gas mixtures will not affect the separation performance."

In supplementary information:

Added:

Supplementary Fig. 5 (page 9)

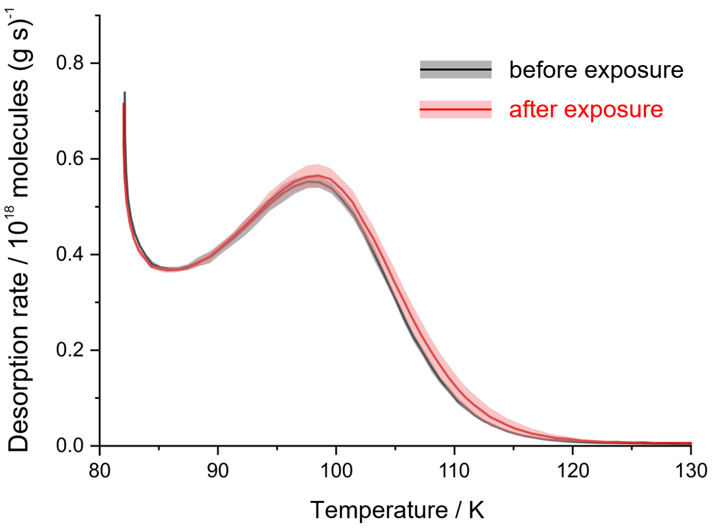

Supplementary Figure 5. **Desorption spectra of D_2 before and after prolonged exposure of Ag(I)-exchanged zeolite to T_2.** Desorption rates during heating at 0.1 K s^{-1} after adsorption at ~ 82 K for 10 min. D_2 was introduced at a pressure of 1 kPa. The lines represent the mean values, the shaded areas show the standard deviations for triplicate TDS records before and after T_2 exposure of the zeolite. For exposure, T_2 was introduced at a pressure of 1 kPa and was left in the adsorption equilibrium at ~ 82 K for 4 h. T_2 was then desorbed in a TDS run. The total number of decays of adsorbed T_2 during exposure was 1.22×10^{16}.

Supplementary Table 3 (page 9)

Supplementary Table 3. **Total uptakes of D_2 on Ag(I)-exchanged zeolite before and after prolonged exposure to T_2.**

**No. of TDS run**	**Gas uptake** (mmol g^{-1})	
	**before exposure**	**after exposure**
1	0.191	0.212
2	0.195	0.194
3	0.199	0.201
**Mean value:**	0.195	0.202
**Standard deviation:**	0.004	0.009

NCOMMS-25-85173A:

Point-by-point response to the Reviewers' comments

We wish to thank all Reviewers for the time and effort they have taken to evaluate our manuscript.

Reviewer#1 (remarks to the author)
The authors have fully addressed my concerns. I guess it can be published now.

Reviewer#2 (remarks to the author)
The authors have made substantial revisions to the manuscript, and all reviewer concerns have been thoroughly and appropriately addressed. Consequently, the manuscript is acceptable for publication in its current form.

Reviewer #4 (remarks to the author)
The revisions made by the authors are satisfactory and successfully address the previous concerns. In my opinion, the manuscript is now ready for publication.

We sincerely thank Reviewers #1, #2, and #4.

Reviewer#3 (remarks to the author)
This revised manuscript presents a compelling and experimentally rigorous validation of Ag-exchanged zeolite Y for the selective separation of hydrogen isotopologues, particularly addressing the long-standing gap in experimental data regarding tritium. The authors have successfully employed TDS to demonstrate exceptional selectivity factors, notably the ST/H value of 244, which significantly outperforms many conventional methods and aligns well with theoretical predictions for chemical affinity quantum sieving. The inclusion of radiation stability tests is a particularly strong addition, effectively mitigating concerns about the radiolytic reduction of Ag(I) sites, a critical factor for practical application in fusion reactor environments. The author has made good revisions to the manuscript and addressed the concerns I raised, and I think that the paper is now ready for publication.

We sincerely thank the Reviewer.

The Minor issues:

1. The abstract reports the ternary mixture enrichment ratio as "1:41:175," whereas the Results section specifies the percentages as "0.5%, 18.9%, and 80.6%," suggesting a precision of one decimal place for the percentages. These two representations are not mathematically consistent.

Answer: We have replaced the percentages with the enrichment ratios to ensure consistency.

2. The binary selectivity for H2/T2 is reported as "244.1" in the Results, but rounded to "244" in the Abstract and Conclusion.

Answer: To ensure consistency, we replaced the selectivity in the Results section with rounded data.
